# Naphthalene diimide–amino acid conjugates as novel fluorimetric and CD probes for differentiation between ds-DNA and ds-RNA

**DOI:** 10.3762/bjoc.16.170

**Published:** 2020-08-19

**Authors:** Annike Weißenstein, Myroslav O Vysotsky, Ivo Piantanida, Frank Würthner

**Affiliations:** 1 Institut für Organische Chemie, Universität Würzburg, Am Hubland, 97074 Würzburg, Germany; 2 Division of Organic Chemistry & Biochemistry, Ruđer Bošković Institute, PO Box 180, 10002 Zagreb, Croatia,; 3 Center for Nanosystems Chemistry (CNC), Universität Würzburg, Theodor-Boveri-Weg, 97074 Würzburg, Germany

**Keywords:** amino acid–fluorophore conjugate, circular dichroism, DNA/RNA recognition, fluorescence, intercalation, naphthalene diimide

## Abstract

Two novel unnatural amino acids, prepared by linking a dicationic purple-coloured and fluorescent naphthalene diimide (NDI) at core position to amino acid side chains of variable length, strongly interacted with ds-DNA/RNA by threading intercalation. Different from a reference NDI dye with identical visible range absorbance (520–540 nm) and Stokes shifts in emission (+60 nm, quantum yield > 0.2), only these amino acid–NDI conjugates showed selective fluorimetric response for GC-DNA in respect to AT(U)-polynucleotides. The DNA/RNA binding-induced circular dichroism (ICD) response of NDI at 450–550 nm strongly depended on the length and rigidity of the linker to the amino acid unit, which controls the orientation of the NDI unit inside within the intercalative binding site. The ICD selectivity also depends on the type of polynucleotide, thus the studied NDI dyes act as dual fluorimetric/ICD probes for sensing the difference between here used GC-DNA, AT-DNA and AU-RNA.

## Introduction

The interplay of non-covalent interactions between nucleic acids and proteins or peptides is the basis of life and is also often used for the design of artificial small molecules, aiming for sensing or control of biorelevant processes. Many naturally occurring bioactive molecules contain a short peptide chain and DNA/RNA interacting aromatic moiety, for instance peptide-based DNA/RNA-intercalators [1–2], as well as many DNA/RNA groove binding small molecules [3–4]. Inspired by these natural examples, the development of novel DNA/RNA targeting synthetic molecules has been in scientific focus for several decades, consequently becoming increasingly complex [5–7]. This includes examples incorporating several types of non-covalent interaction with DNA/RNA (intercalation, groove binding, positive–negative charge interaction [5,8]) in one molecule or even modified biomacromolecules (e.g., proteins [9]). One of the approaches relies on amino acids conjugated with various DNA/RNA-binding chromophores, thereby yielding effective spectrometric sensing systems due to their interactions with DNA. In this approach, chromophores can be combined with peptide sequences in various ways, thus giving access to large libraries of close analogues. Further, in such peptide-based chromophore systems, a multitude of different chromophores/fluorophores [10] could allow fine tuning of spectroscopic responses to various DNA/RNA sequences. With this concept in mind, Piantanida and co-workers recently developed several series of fluorophore–amino acid conjugates, thereby making use of the availability of C- and N-terminal amino acid residues for peptide-bond formation. Also, several short multichromophoric peptide constructs were prepared and studied with regard to their interactions with DNA/RNA [11–16].

These inspiring results encouraged us to broaden the palette of available amino acid (AA)–chromophore conjugates. Therefore, in this work we have chosen a naphthalene diimide (NDI) chromophore [17–20], a well-known DNA/RNA binding moiety, which differs from previously used dyes by its ability to intercalate into ds-DNA/RNA by “threading” through the polynucleotide double helix [21–22]. Such “threading intercalation” indicates that a large aromatic moiety with bulky groups at opposite ends is inserted between two DNA or RNA base pairs, whereby bulky substituents end in both, the minor and major groove of the polynucleotide. Such bulky groups positioning requires the DNA double helix to shortly open at a binding site and close upon threading intercalator insertion. Also, the chosen NDI chromophore is characterised by easily tuneable emission wavelengths [23], and therefore adaptable for the design of FRET pairs with other dyes along the peptide backbone. Such amino acid construct would bring a novel functional property into peptide–multichromophore systems targeting DNA/RNA.

**Figure 1:**
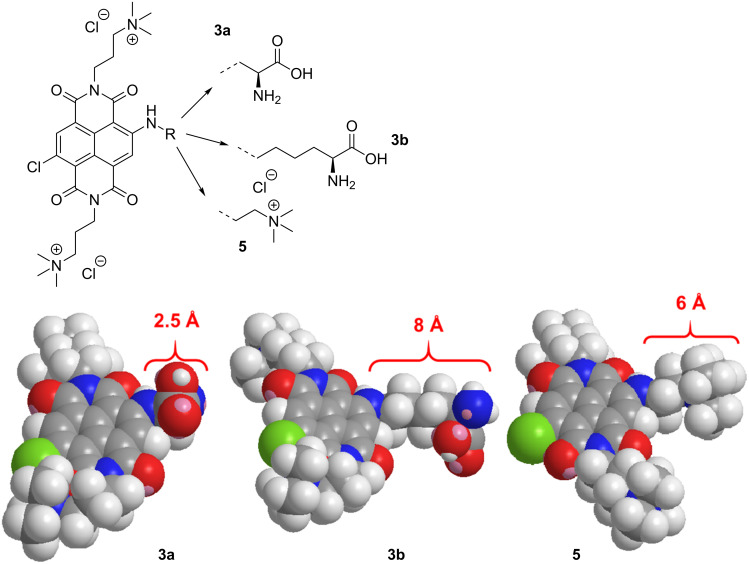
Structures of investigated compounds stressing steric differences in linker length attached to the NDI core.

For the design of our new constructs we noticed that the majority of DNA/RNA targeting NDIs is substituted at the imide positions, although such substituted NDI derivatives tend to hydrolyse at the imide positions in basic aqueous environment [24]. It is therefore important during the synthesis and processing to work under acidic to neutral conditions to ensure the stability of the molecules. In addition to the pH value, the hydrolysis also depends on the position of cationic ammonium groups, namely, the distance of the charged group from the imide position is proportional to its stability [24]. However, too long side chains can interfere with threading intercalation into ds-DNA/RNA. As a compromise, a 3-trimethylammoniumpropyl substituent was uniformly introduced at both imide positions (Figure 1). As shown previously by Würthner and co-workers, amine and halogen substituents at the NDI 2,6-positions have a remarkable effect on the NDI chromophore, as they endow the otherwise colourless and non-fluorescent core-unsubstituted NDI with a new charge transfer band with an absorption maximum in the visible spectral range and a high fluorescence quantum yield (up to 58%) [23,25]. Further, the 2-amino substituent offers the possibility to connect various amino acid side chains, thus preparing novel fluorophore–amino acid (AA) conjugates. The amino acids (*S*)-2,3-diaminopropionic acid (ʟ-Dap) and (*S*)-2,6-diaminohexanoic acid (ʟ-Lys) were chosen to test the difference in the aliphatic linker lengths (Figure 1) on DNA/RNA binding. For comparison purposes, reference compound **5** with 2-(trimethylammonium)ethylamine instead of an amino acid (Figure 1) was prepared.

In our study of the interactions with DNA/RNA weakly acidic conditions (pH 5) were chosen to complement available pH-dependent AA-fluorophores used in our previous research (PHEN-AAs [12,26] and GCP-derivatives [15,27]), which allowed pH control over DNA/RNA binding. There are several other systems also taking advantage of pH-controlled DNA binding [28], and such pH control could be further used also in selective antitumour strategies, taking advantage of many solid tumours having significantly lowered extracellular pH [29–30]. That would allow in future research combining of the here studied NDI–AA derivatives with the above mentioned and other pH-sensitive fluorophore–AA, to prepare multicolour spectroscopic probes and eventually also FRET pairs.

Also, the two NDI–AA conjugates **3a** and **3b** have protonatable amino groups at the amino acid side chain, which should be fully protonated at pH 5, thus affording three positive charges resembling the reference compound **5**.

## Results and Discussion

### Synthesis

**Scheme 1:**
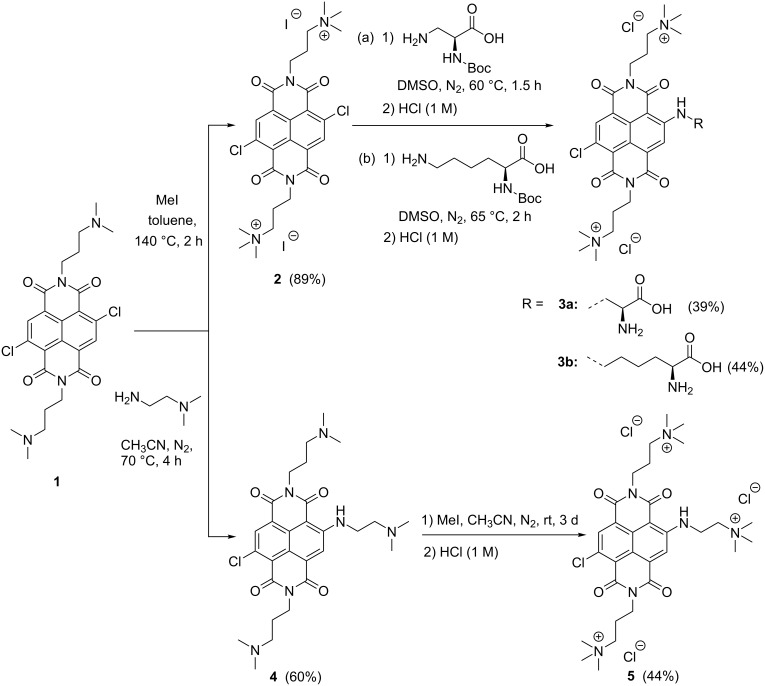
Synthesis of water-soluble naphthalene diimides **3a**,**b**, and **5**.

Based on the design concept discussed above, the NDIs **3a**,**b** and **5** were obtained in three synthetic steps according to Scheme 1, starting from the literature-known dichloro NDI **1** having two 3-dimethylaminopropyl groups attached to the imide nitrogens [31]. This compound was first methylated at the nitrogen atoms by the reaction with iodomethane in refluxing toluene, giving the diammonium NDI **2** in a very good yield of 89%. In the second step, the naphthalene nucleus was functionalized via a nucleophilic aromatic substitution with the Boc-protected amino acids ʟ-Dap and ʟ-Lys. The reaction was carried out in dry DMSO at 60–65 °C for 1.5–2 hours. After two preparative HPLC purifications in an acidic environment (with TFA) and treatment with 1 M HCl solution the Boc protecting group was split off (third step), and the desired NDI derivatives **3a** and **3b** were obtained in a yield of 39% and 44%, respectively. For the preparation of NDI **5**, compound **1** was first monofunctionalized at the core with 2-dimethylaminoethylamine in a nucleophilic substitution and then methylated. NDI **4** could be isolated in a yield of 60% after purification by column chromatography. In the last synthetic step, the molecule was triply methylated with iodomethane in acetonitrile at room temperature for three days. After complete methylation, purification by preparative HPLC and treatment with 1 M HCl solution, NDI **5** with three cationic substituents at the imide and bay positions could be obtained in 44% yield. The ^1^H, ^13^C NMR data, and high-resolution mass spectra correspond well with the structures of all new compounds synthesised.

### Spectrophotometric properties of the water-soluble NDIs

**Figure 2:**
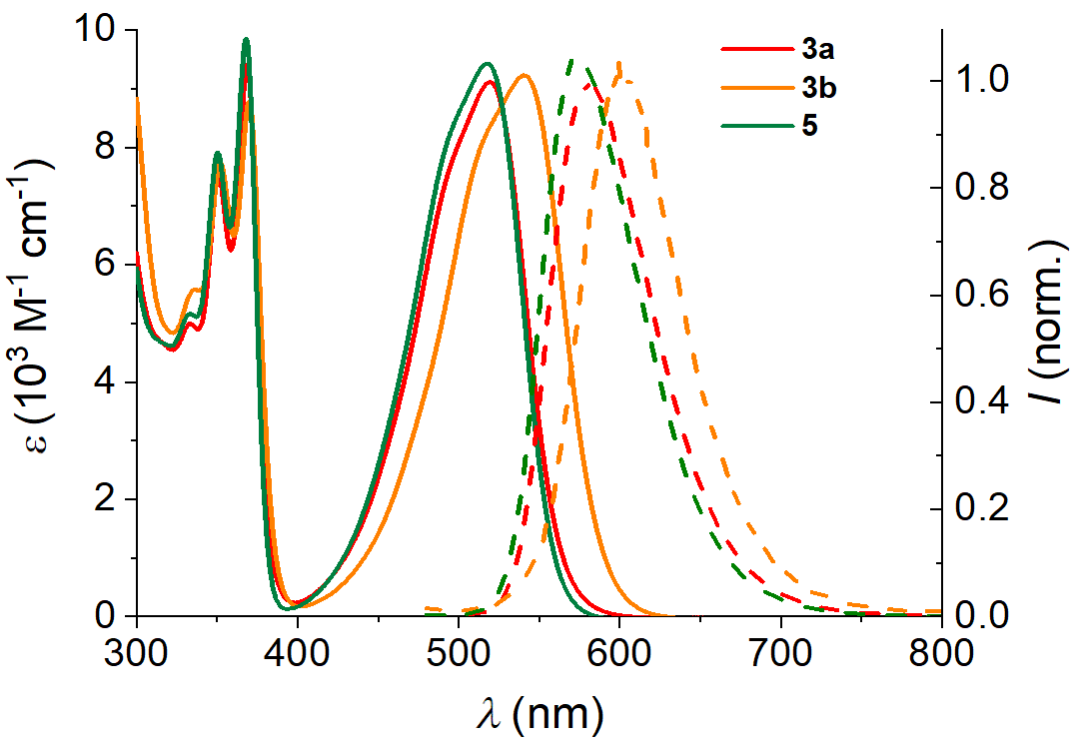
UV–vis absorption (solid line) and fluorescence spectra (dashed line) of NDI **3a**,**b**, and **5** (*c* = 4.5 × 10^−5^ M in cacodylate buffer at pH 5.0, λ_ex_ = 470 nm) at 23 °C.

The spectrophotometric properties of the NDIs **3a**,**b**, and **5** were investigated in cacodylate buffer at pH 5.0 for easier comparison with complementary pH-dependent AA–fluorophores used in our previous research (PHEN–AAs [26] and GCP derivatives [27]). Additionally, the stability of similar NDI compounds is greater in weakly acidic buffer solution over a longer period of time [24]. Compounds **3a**,**b**, and **5** revealed absorption maxima of 518–540 nm with molar extinction coefficients of nearly 10000 M^−1^ cm^−1^ (Figure 2). Further, **3a**,**b**, and **5** show strong fluorescence at 573–602 nm with significant Stokes shifts (+60 nm) and quantum yields of 10–32% (Table 1).

**Table 1:** Optical properties of **3a**,**b**, and **5** in cacodylate buffer (pH 5.0) at 23 °C.

NDI	λ_abs_ [nm]	ε [M^−1^ cm^−1^]	λ_em_^a^ [nm]	Φ_F_

**3a**	519	9100	581	0.21
**3b**	540	9200	602	0.10
**5**	518	9400	573	0.32

^a^Excitation wavelength: λ_ex_ = 470 nm to avoid overlap with emission spectrum.

### Theoretical calculations

To get insight into the electronic and optical properties of the 2-amino-6-chloro-substituted NDIs, we carried out computational investigations by using the Gaussian 09 program suite [32]. In particular, we restricted our study to the simplest and general case of the chloro-methylamino-substituted compound Cl-NDI-NMe by cutting the aliphatic chain substituents in the imide positions and considering a methylamino substitution on the core. This simplification is supported by the assumption that the substituents in the imide positions and on the nitrogen on the core are known to have a negligible effect on the chromophore, since both the HOMO and the LUMO have a node at these positions [33].

**Figure 3:**
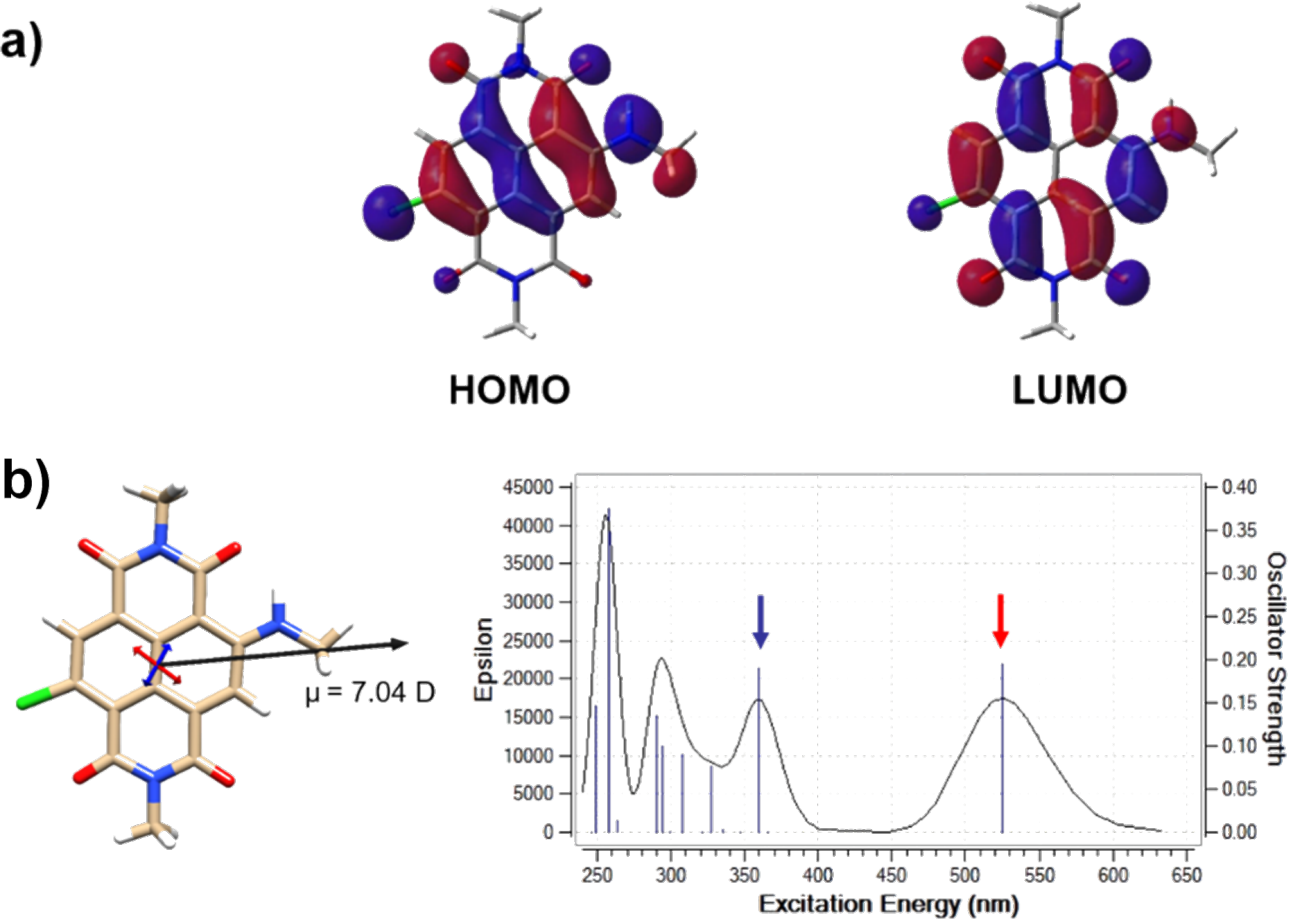
Calculations for Cl-NDI-NMe model compound (at the B3LYP/6-31+G** level of theory) in water (PCM). a) HOMO and LUMO molecular orbitals (isovalue surface 0.03 au). b) The molecular ground state dipole moment is indicated by the black arrow. The transition dipole moments calculated by TD-DFT (at the B3LYP/6-31+G** level of theory) for the lowest transitions in the visible range of the spectrum (indicated in the spectra by the red and blue arrows) are shown respectively on the structure.

Geometry optimization by DFT method (at the B3LYP/6-31+G** level of theory) confirmed a planar and rigid NDI chromophore in the ground state (Figure 3). The predicted HOMO and LUMO show remarkable (HOMO) and modest (LUMO) delocalization on the core-substituents which suggest an important contribution of the chlorine atom to the optical features and chemical reactivity [23]. Additionally, the molecule features a high molecular ground-state electrical dipole (µ = 7.04 D), which is an evidence for an intramolecular charge transfer (CT) character of the chromophore [34]. The predicted UV–vis spectra by TD-DFT are in excellent agreement with the experimental one (Figure 3, Table 1, the vibrational coupling is neglected). The transition dipole moment associated with the HOMO-to-LUMO transition in the visible range (red arrow) and with the higher energy transition (blue arrow) are respectively shown superimposed on the minimised structure.

### Interactions of **3a**,**b**, and **5** with ds-DNA/RNA

For a study of interactions with polynucleotides we have chosen synthetic long ds-DNA: poly(dG-dC)_2_ and poly(dA-dT)_2_ and ds-RNA: poly(A)-poly(U), as well as mixed sequence ct-DNA (48% of G-C base pairs). The reason for the use of long polynucleotides was to avoid the physiologically non-relevant interactions with a short duplex oligonucleotide: for instance, binding of a large aromatic dye by aromatic stacking on the terminal base pairs [35], as a competitive binding mode to expected intercalation. Further, the chosen DNA/RNA polynucleotides are characterised by different secondary structures [36–37]: poly(dA-dT)_2_ representing the B-helical structure with accessible minor groove at variance to poly(dG-dC)_2_, which has sterically hindered minor grooves by amino groups, and poly(A)-poly(U) is an A-helix with major groove available for binding of bulky small molecules [38]. The interactions of the NDI derivatives **3a**,**b**, and **5** with DNA/RNA were examined first by means of the thermal denaturation method.

Various double-stranded DNA or RNA are known upon heating to dissociate into two single-stranded polynucleotides at a characteristic well-defined temperature (*T*_m_ value). A non-covalent interaction of small molecules usually increases the thermal stability of the ds-polynucleotide, consequently causing an increase of the denaturation temperature (∆*T*_m_). This ∆*T*_m_ value can be related to the various binding modes of a small molecule to DNA/RNA [39]. The studies with poly(dG-dC)_2_ were not possible due to the high melting temperature of >100 °C.

**Figure 4:**
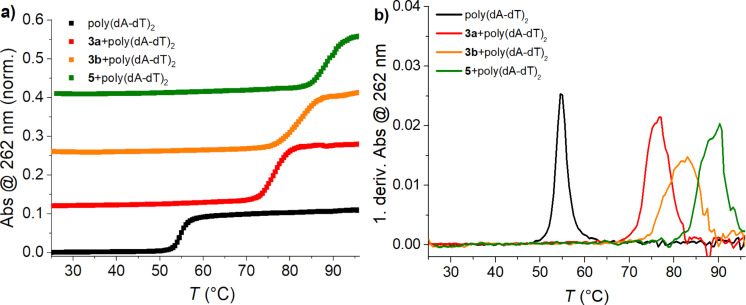
(a) Melting curve of poly(dA-dT)_2_ alone and after the addition of NDI **3a**,**b**, and **5** (*r* = 0.3 ([NDI]/[polynucleotide])) (sodium cacodylate buffer, pH 5.0, ionic strength (I) = 0.05 M). (b) First derivative function of absorption from temperature.

As shown in Figure 4 and Table 2, the addition of all studied compounds resulted in very strong stabilisation effects on both, poly(dA-dT)_2_ and poly(A)-poly(U). This similarity of stabilisation of ds-DNA and ds-RNA supports the presence of an intercalative binding mode because DNA groove binders are usually strongly selective toward DNA [8]. The NDI **5** caused by far the strongest stabilisation – likely due to the three permanent charges. NDI **3b**, with a longer and thus more flexible linker, has a higher ∆*T_m_* value than the shorter and less adaptable NDI **3a**.

**Table 2:** ∆*T*_m_ values (°C) of the investigated polynucleotides when **3a**,**b**, and **5** were added in the ratio *r* = 0.3 (sodium cacodylate buffer, pH 5.0, I = 0.05 M).

polynucleotide	*r* =^a^	**3a** ∆*T*_m_ [°C]^b^	**3b** ∆*T*_m_ [°C]^b^	**5** ∆*T*_m_ [°C]^b^

poly(A)-poly(U)	0.3	+17.5	+25.1	+30.3
poly(dA-dT)_2_	0.3	+21.6	+28.2	+35.4

^a^*r* = [NDI]/[polynucleotide]; ^b^error in ∆*T*_m_: ± 0.5 °C.

For an accurate determination of the binding affinity we took advantage of the fluorescence of **3a**,**b**, and **5**. Since suspected threading intercalation usually requires longer incubation times, the time required for reaching equilibrium was checked by repeatedly collecting emission spectra upon additions of DNA or RNA aliquots to the dye solution. Accordingly, an incubation period of 180 s proved to be sufficient.

**Figure 5:**
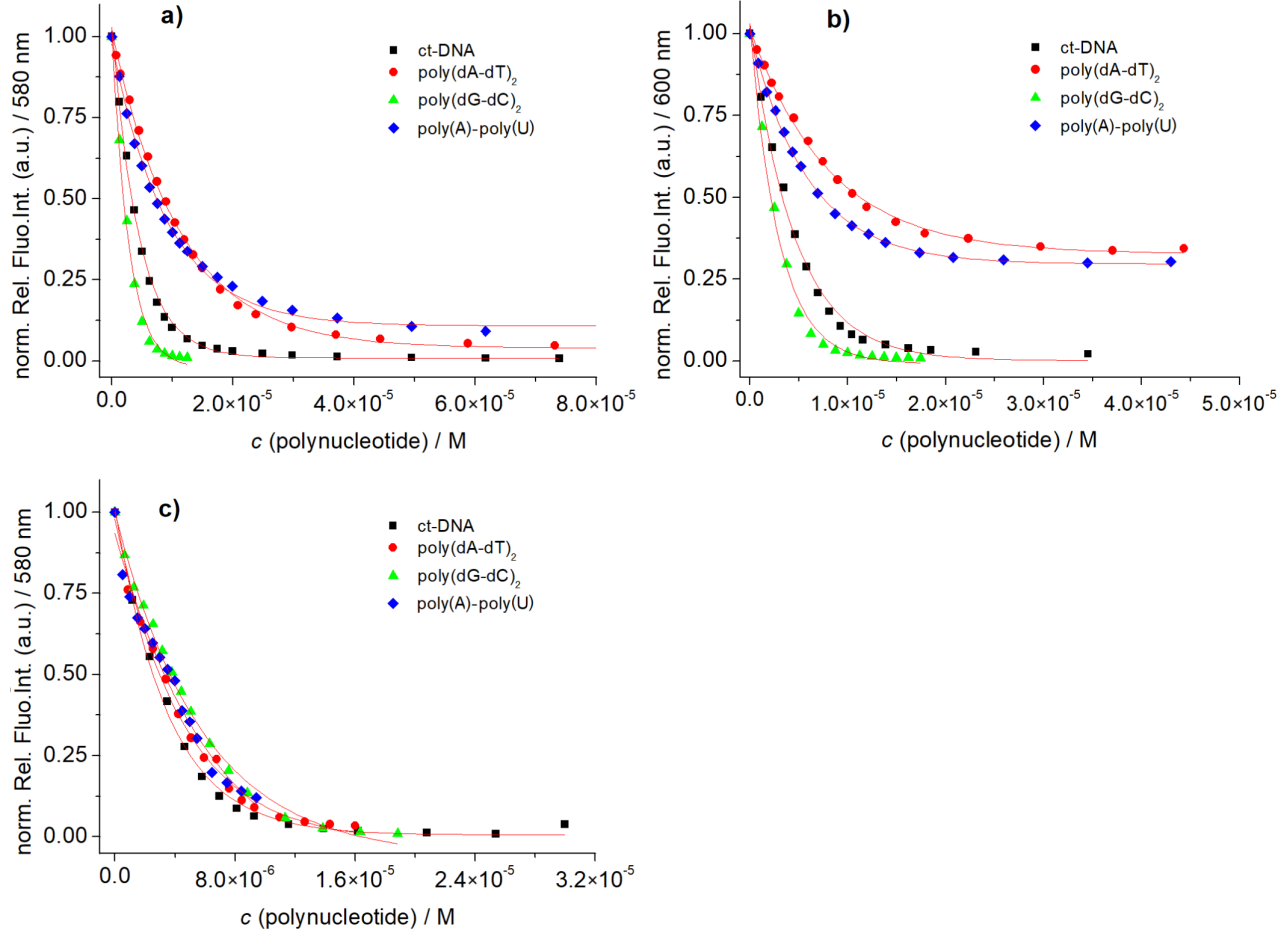
Changes in fluorescence intensity (spectra are normalized) of (a) **3a** (*c* = 1.0 × 10^−6^ M), (b) **3b** (*c* = 1.0 × 10^−6^ M) and (c) **5** (*c* = 1.0 × 10^−6^ M) when adding polynucleotides at 25 °C (cacodylate buffer, pH 5.0, I = 0.05 M) (λ_ex_ = 470 nm). Red lines denote non-linear fittings to experimental data by means of the Scatchard equation [40–42].

Generally, the addition of any DNA/RNA resulted in a strong quenching of **3a**,**b**, and **5** emission. However, the emission of reference compound **5** was non-selectively quenched by any DNA/RNA (Figure 5c), whereas solutions of **3a** or **3b** showed a stronger quenching for GC-DNA and the weaker for AT(U)-polynucleotides (Figure 5a and b). Because guanine is the most electron-rich nucleobase, this behaviour points at a fluorescence quenching mechanism by charge transfer from the electron-rich purine bases to the electron-poor NDI molecular probes.

Processing the titration data by means of non-linear curve fitting to the Scatchard equation [40–42], yielded binding constants log*K*_s_ and binding ratios *n*_[bound NDI]/[polynucleotide]_ (Table 3). Although all binding constants are still in the same range (log*K*_s_ = 6–7), our comparison revealed for compounds **3a** and **3b** a clear preference toward GC-DNA in respect to AT(U) sequences. We assume that this is due to the fact that the more electron-rich guanines quench the fluorescence most efficiently via charge transfer interactions with the NDI molecular probes, similar as noted for specific guanine-induced emission quenching of acridine or 4,9-diazapyrene derivatives [43].

**Figure 6:**
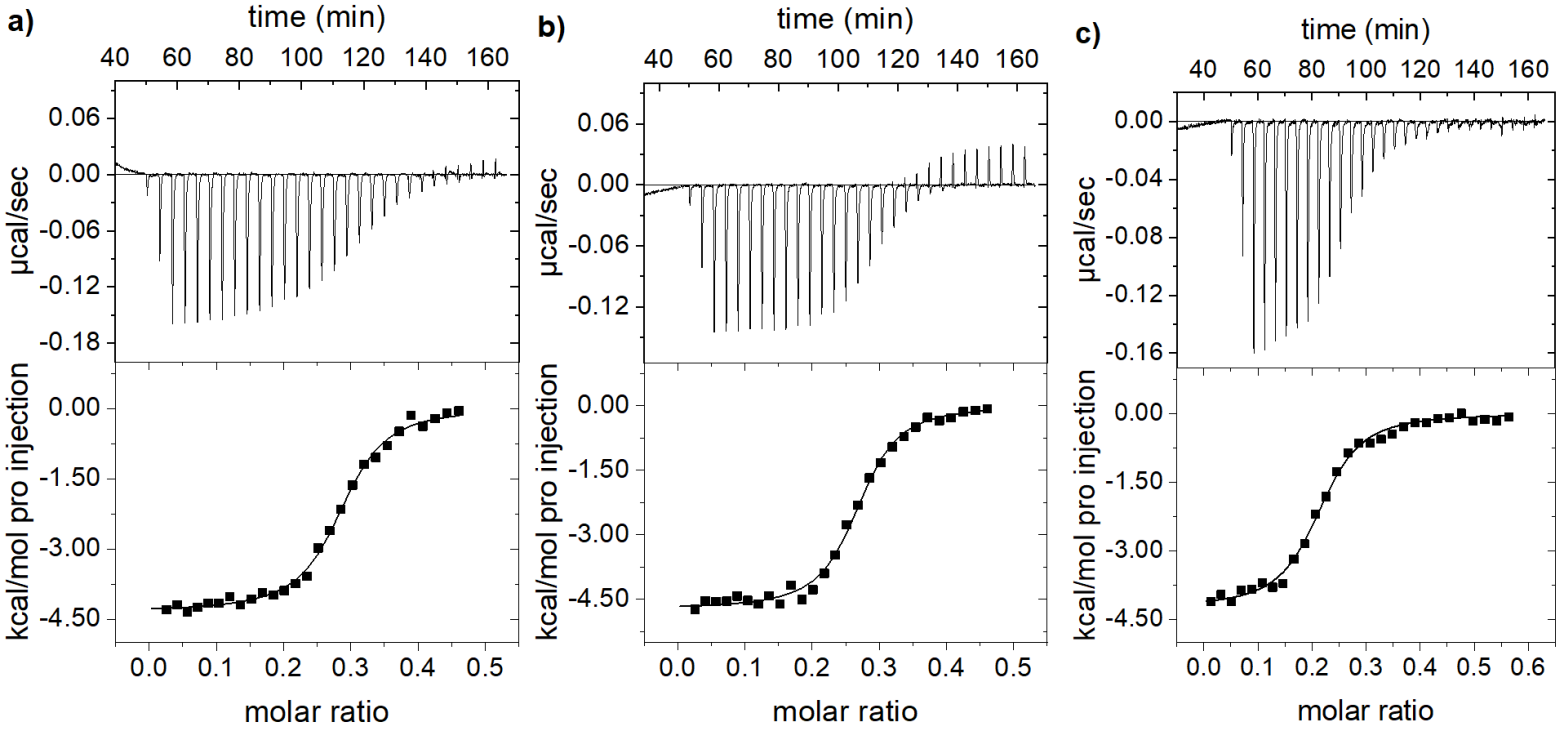
Calorimetric titration of a poly(dG-dC)_2_ solution in sodium cacodylate buffer (pH 5.0) at 298 K with NDI (a) **3a**, (b) **3b**, and (c) **5**. Top: data obtained after periodic injection of a NDI solution. Bottom: graphs of heat/mol released per injection versus the molar ratio of NDI to polynucleotide. Black lines denote non-linear fitting to experimental data by one binding site model, in agreement with the Scatchard equation.

In order to confirm the fluorimetric data by an independent method, and also to characterise thermodynamic parameters of complex formation, ITC titrations were performed (Figure 6, Supporting Information File 1, Figures S28–S30), and the results are summarised in Table 3. The values of log*K*_s_ obtained from fluorimetric and ITC experiments were generally in good agreement and minor differences within the same order of magnitude. Also, our ITC titrations revealed for all dye–polynucleotide complexes similar sets of negative enthalpy (Δ*H* [kcal/mol]) and positive entropy (Δ*S* [cal/mol/K]) values, pointing out that the complexation processes are enthalpy-driven and characterised by the same type of binding mode, i.e., intercalation [17,44]. The relatively large entropy contribution might be attributed to the displacement of cations and water molecules from the DNA/RNA grooves [44–45] by side arms interacting with the grooves and thereby supporting the binding.

**Table 3:** Binding constants (log*K*_s_) and stoichiometry *n*_([bound NDI]/[polynucleotide])_ calculated by non-linear fitting of fluorescence^a^ and ITC^b^ titrations of **3a**,**b**, and **5** (*c* = 1.0 × 10^−6^ M) with ct-DNA, poly(A)-poly(U), poly(dA-dT)_2_, and poly(dG-dC)_2_ at 25 °C (cacodylate buffer, pH 5.0, I = 0.05 M).

	ct-DNA			poly(A)-poly(U)			poly(dA-dT)_2_			poly(dG-dC)_2_		
	*I*/*I*_0_^c^ Δ*H*	*n* Δ*S*	log*K*_s_^a^ log*K*_s_^b^	*I*/*I*_0_^c^ Δ*H*	*n* Δ*S*	log*K*_s_^a^ log*K*_s_^b^	*I*/*I*_0_^c^ Δ*H*	*n* Δ*S*	log*K*_s_^a^ log*K*_s_^b^	*I*/*I*_0_^c^ Δ*H*	*n* Δ*S*	log*K*_s_^a^ log*K*_s_^b^

**3a** ITC data^b^	0.00 −4.22	0.17 14.1	**6.8** 6.2	0.21 −2.33	0.20 19.2	**6.0** 5.9	0.04 −3.85	0.07 16.0	**6.0** 6.3	0.00 −4.33	0.26 16.0	**7.2** 6.7
**3b** ITC data^b^	0.00 −4.26	0.15 15.5	**6.7** 6.5	0.25 −4.97	0.16 13.4	**6.4** 6.6	0.31 −3.46	0.10 19.4	**6.1** 6.8	0.00 −4.72	0.23 15.0	**6.6** 6.7
**5** ITC data^b^	0.00 −4.41	0.21 14.1	**7.1** 6.4	0.00 −2.18	0.14 23.3	**6.2** 6.7	0.00 −2.78	0.22 20.0	**6.6** 6.4	0.00 −4.23	0.14 15.5	**6.4** 6.5

^a^Titration data were processed using the non-linear curve fitting using Scatchard equation [40] gave ratios of *n*_[bound NDI]/[polynucleotide]_ = 0.1–0.3. For easier comparison, all log*K*_s_ values were recalculated for fixed *n* = 0.2. Correlation coefficients were >0.99 for all calculated *K*_s_ values; ^b^ITC titrations were performed at 298 K (sodium cacodylate buffer, pH 5.0, I = 0.05 M) and fitted to “One Set of Sites” binding model [46–48] for fixed *n* = 0.2; giving enthalpy (Δ*H* [kcal/mol]) and entropy (Δ*S* [cal/mol/K]); ^c^fluorescence of the NDI–polynucleotide complex (*I*) and initial fluorescence (*I*_0_) of the NDI calculated according to the Scatchard equation.

### Circular dichroism experiments

**Figure 7:**
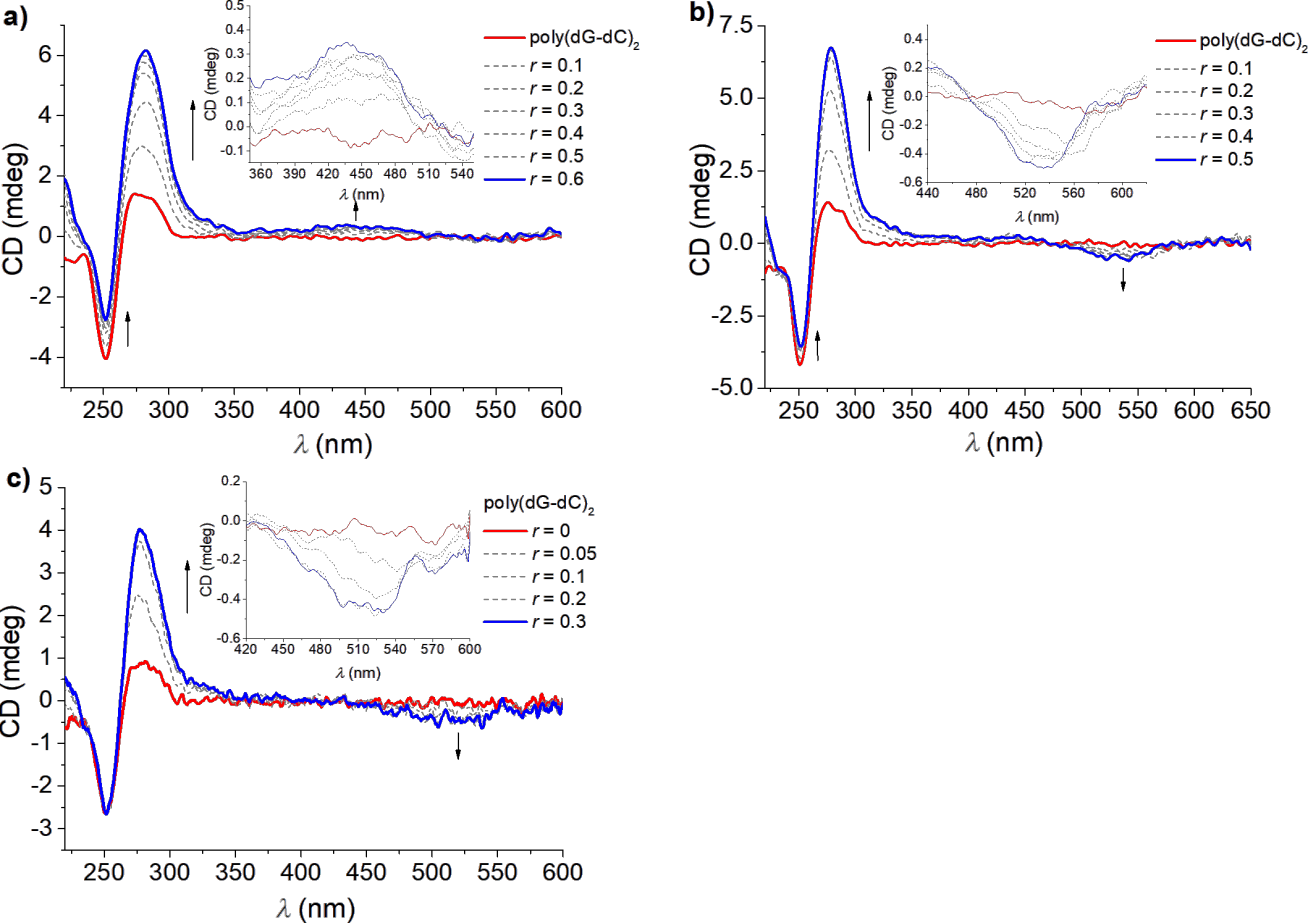
CD titration of poly(dG-dC)_2_ (*c* = 2.0 × 10^−5^ M) with (a) **3a**, (b) **3b**, and (c) **5** with increasing molar ratios of *r* = [NDI]/[polynucleotide] at 25 °C (cacodylate buffer, pH 5.0, I = 0.05 M).

CD spectroscopy is an ideal method to get insight into the changes of the polynucleotide secondary structure upon binding of small molecules [49–50]. Also achiral small molecules can eventually acquire induced CD (ICD) upon binding to chiral polynucleotides, which could give useful information about modes of interaction [49–50]. The NDIs **3a**,**b** are chiral but the chirality of the amino acid residue is not transferred through the aliphatic linker to the NDI core, since these NDI derivatives do not show intrinsic CD spectra in the range of NDI absorbance (230–600 nm). Titrations of all ds-DNA/RNA with any of our NDIs resulted in a strong increase of the bands at 270–290 nm (Figure 7 and Supporting Information File 1), which are commonly attributed to nucleobase pairs. However, since it is not likely that the chirality of the double helix will strongly increase upon binding of a small molecule, the most prominent changes at 300 nm and above are most likely attributable to ICD bands of the NDI core bound to the polynucleotide in a uniform orientation related to the DNA/RNA chiral axis [49–50].

**Figure 8:**
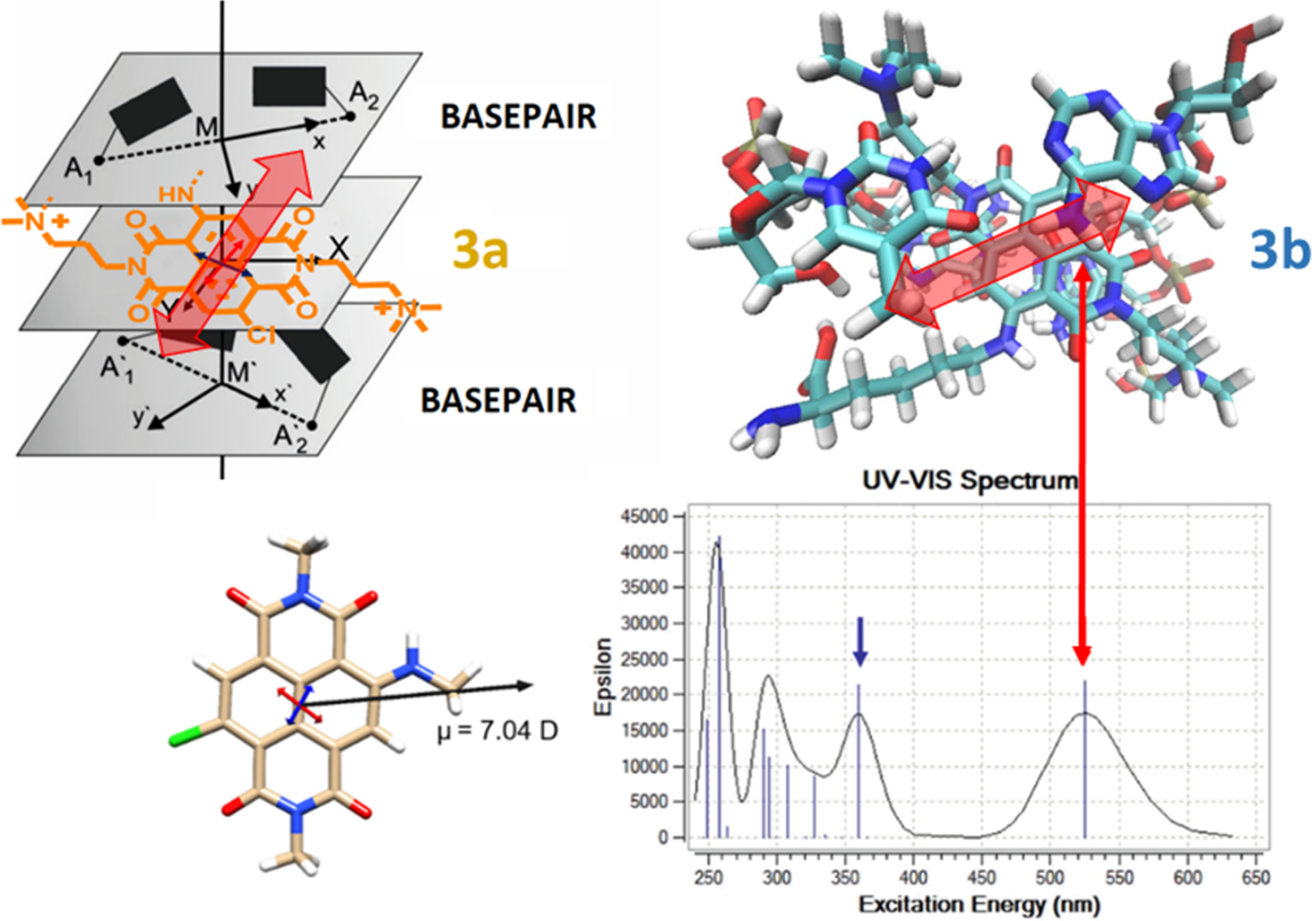
Schematic representation of the alignment of the intercalating **3a** (left) and **3b** (right) between the base pairs of the oligonucleotide. The complex with **3b** was prepared analogously to the NDI analogue [53] by replacing the threading intercalator in PDB258D [54] with **3b**, and performing MM2 minimisation in vacuum. Bottom: The orientation of transition dipole moments (red arrow for 450–600 nm range) according to the calculations made for the spectra shown in Figure 2.

Furthermore, differences in the ICD response in the wavelength range from 400 to 540 nm were observed between our NDI compounds which were most pronounced upon binding to poly(dG-dC)_2_ (Figure 7). These simple and rather weak ICD signals, along with strong thermal stabilisation (Table 2) and high affinity (Table 3) strongly support intercalation of individual NDI molecules between the base pairs of the ds-DNA [51–52]. Here, their opposite sign (for **3a** positive ICD, for **3b** and **5** negative ICD) points to different orientations of the NDI transition dipole moment with respect to the DNA chiral axis [50]. A positive sign observed for **3a** suggests that the long axis of the NDI chromophore is perpendicular to the longitudinal axis of the base pairs (red arrow in Figure 8, for **3a**), while a negative one observed for **3b** suggests a parallel arrangement of the NDI dye to the base pairs (red arrow in Figure 8, for **3b**).

The complexation with poly(dA-dT)_2_ (Supporting Information File 1, Figure S27) resulted in a negative ICD band (505 nm) only for compound **5**, whereas **3a** and **3b** did not show any measurable ICD signal, likely due to the intercalation of the NDI chromophore at approximately 45° with respect to the base pair longer axes, thus yielding negligible intensity of the ICD bands [49–50]. A mixed sequence ct-DNA (48% of GC-base pairs) induced for all three dyes negative ICD signal (Supporting Information File 1, Figure S25), indicating predominantly a parallel orientation for all dyes as shown in Figure 8 for the DNA–**3b** complex. The absence of any measurable ICD signal for poly(A)-poly(U) (ds-RNA) (Supporting Information File 1, Figure S26) supports the intercalation of all NDI chromophores at approximately 45° with respect to the base pair longer axes, thus yielding negligible intensity of ICD bands [49–50]. Such different sets of ICD band responses, which varied not only with respect to differences in the dye structure but also depended strongly on the DNA/RNA secondary structure revealed a high sensitivity of the studied NDI–polynucleotide systems, thereby providing insight into the aromatic core position within the intercalative binding site.

## Conclusion

The new amino acid conjugates **3a**, **3b**, and reference compound **5** bearing the fluorescent NDI tag molecules showed moderate absorbance in the mid-visible range (λ_abs_ 520–540 nm) and significant Stokes shifts of emission (+60 nm) characterised by good quantum yield in aqueous solution. Thus, NDIs **3a** and **3b** are novel intensively fluorescent non-natural amino acid probe molecules with both, N- and C-termini available for incorporation into any peptidoid construct requiring a fluorescent tag.

All studied compounds strongly interact with similar affinity (log*K_s_* 6–7) with ds-DNA/RNA by intercalation (as confirmed by high thermal stabilisation and CD results), and since intercalation of NDI is only possible by passing one bulky substituent through the polynucleotide double helix, all studied molecules can be regarded as threading intercalators. Complexes with DNA/RNA are additionally stabilised by interactions of positively charged side chains. The spectrophotometric response of these compounds showed pronounced differences and was in some cases highly sensitive on ds-polynucleotide composition and secondary structure. Thus, reference **5** with three permanently charged aliphatic sidechains was non-selective, giving virtually the same fluorimetric and CD response for all DNA/RNA. In contrast, the introduction of amino acid side chains in **3a** and **3b** yielded selective fluorimetric responses between GC and AT(U)-polynucleotides. Moreover, the length and rigidity of the linker to the amino acid unit controlled the positioning of the NDI core inside the intercalative binding site: in GC-DNA, **3a** (shorter, more rigid) afforded an ICD band of opposite sign as observed for **3b** or **5**. This ICD selectivity also depended on the type of polynucleotide, thus we learned that some core-functionalized NDI dyes can directly report the difference between here used GC-DNA, AT-DNA, and AU-RNA.

Since till now various NDI derivatives were applied for binding and sensing different types of DNA/RNA constructs, including G-quartets [55–57], and other, more complex sequences, the herein presented amino acid–NDI conjugates may in future also be investigated for such applications, either directly or incorporated in peptidoid constructs. Indeed, the colourful and fluorescent NDIs **3a** and **3b** are ideal for use in peptide-backbone constructed multichromophores targeting FRET-based sensing [14,26,58]. For the application of here presented results in bioanalytical sciences or biologically relevant studies it will be necessary to further modify the presented compounds and precisely collect information about their sensitivity to particular target, read-out accuracy, limits of detection, and selectivity at biorelevant conditions.

## Experimental

All solvents were purchased from commercial sources and used as received. Solvents for spectroscopic studies were of spectroscopic grade. Polynucleotides poly(dA-dT)_2_, poly(AU), calf thymus (ct)-DNA, and poly(dG-dC)_2_ were obtained from Sigma–Aldrich. The starting compound **1** was prepared according to the literature [31]. Column chromatography was performed on silica gel (MerckSilica 60, particle size 0.04–0.063 mm). Semipreparative HPLC was performed on a Jai system (LC-9105) with a UV–vis detector (UV 3702). The melting points (mp) of compounds were determined with an Olympus BX-41 polarization microscope equipped with a Linkam THMS 600 hot stage and a temperature controller unit. ^1^H and ^13^C NMR spectra were recorded in CD_3_OD, CDCl_3_, D_2_O or DMSO-*d*_6_ at 298 K on a Bruker Avance 400 spectrometer. The chemical shifts are reported in ppm and refer to the residual proton signal of the solvent as internal standard. Signal multiplicities are denoted as s (singlet), d (doublet), t (triplet), and m (multiplet). High-resolution ESI-TOF mass spectrometry was carried out on a MicroTOF focus instrument (BrukerDaltronik GmbH). Lyophilisation dryings were carried out using an ALPHA 2-4 LD device from Martin Christ Gefriertrocknungsanlagen GmbH. Only demineralised water (bidistilled water, Milli-Q) was used as the solvent.

**Synthesis of *N,N*'-bis((3-(trimethylammonium)propyl)amino)-2,6-dichloro-1,4,5,8-naphthalenetetracarboxylic acid diimide diiodide (2):** To a solution of *N*,*N*'-bis((3-(dimethylamino)propyl)amino)-2,6-dichloro-1,4,5,8-naphthalenetetracarboxylic acid diimide (**1**, 1.00 g, 1.98 mmol) in toluene (90 mL) was added iodomethane (2.27 g, 1.00 mL, 16.0 mmol). The reaction mixture was stirred at 130 °C for 2 h. After cooling to room temperature, the resulting light-brown precipitate was collected by filtration, washed with diethyl ether, and dried in vacuum (1.39 g, 89%). Mp 169–174 °C; ^1^H NMR (DMSO-*d*_6_, 400 MHz) 8.61 (s, 2H), 4.12 (t, ^3^*J* = 6.5 Hz, 4H), 3.46 (m, 4H), 3.04 (s, 18H), 2.14 (m, 4H); ^13^C NMR (DMSO-*d*_6_, 101 MHz) 161.1, 160.7, 137.6, 134.1, 127.0, 126.3, 122.5, 63.2, 52.2, 37.7, 21.4; HRMS-ESI^+^ (methanol, *m*/*z*): [M]^2+^ calcd for C_26_H_32_Cl_2_N_4_O_4_, 267.0900; found, 267.0900; UV–vis (MeOH) λ [nm] (ε [M^−1^ cm^−1^]) 397 (10300), 377 (10900), 357 (16000).

**Synthesis of (S)-*N,N*'-bis((3-(trimethylammonium)propyl)amino)-2-(2-amino-2-carboxyethyl)amino-6-chloro-1,4,5,8-naphthalenetetracarboxylic acid diimide dichloride (3a):** Diimide (**1**, 100 mg, 190 µmol) and 3-amino-(*tert*-butoxycarbonyl)-ʟ-alanine (114 mg, 560 µmol) were placed under nitrogen, and dry DMSO (5.0 mL) was added. The reaction mixture was stirred for 1.5 h at 60 °C. After removal of the solvent under reduced pressure, the residue was purified by preparative HPLC using a C-18 reversed-phase column (CH_3_CN/H_2_O/TFA = 25:75:0.1). HCl solution (1 M) was added and the solvent was removed by freeze drying yielding a pink solid (50.0 mg, 39%). Mp 275–279 °C; ^1^H NMR (CD_3_OD, 400 MHz) 8.59 (s, 1H), 8.49 (s, 1H), 4.47 (m, 1H), 4.30 (m, 6H), 3.57 (m, 4H), 3.16 (s, 18H), 2.28 (m, 4H); ^13^C NMR (CD_3_OD, 101 MHz) 169.8, 167.0, 163.4, 163.3, 162.5, 152.7, 135.2, 134.2, 129.1, 128.8, 125.1, 123.1, 122.8, 121.0, 102.7, 65.6, 53.7, 53.7, 53.6, 53.5, 43.6, 39.0, 38.4, 23.2, 23.1; HRMS-ESI^+^ (methanol, *m*/*z*): [M]^2+^ calcd for C_29_H_39_ClN_6_O_6_, 301.1310; found, 301.1317; UV–vis (cacodylate buffer, pH 5.0) λ [nm] (ε [M^−1^ cm^−1^]) 519 (9100), 369 (9400), 351 (7500), 333 (5000); fluorescence (cacodylate buffer, pH 5.0, λ_ex_ = 470 nm): λ_max_ [nm] = 581; Φ_fl_ = 0.21.

**Synthesis of (S)-*N,N*'-bis((3-(trimethylammonium)propyl)amino)-2- (5-amino-5-carboxypentyl)amino-6-chloro-1,4,5,8-naphthalenetetracarboxylic acid diimide dichloride (3b):** Diimide (**1**, 100 mg, 190 µmol) and *N*_α_-(*tert*-butoxycarbonyl)-ʟ-lysine (95.0 mg, 390 µmol) were placed under nitrogen, and dry DMSO (5.0 mL) was added. The reaction mixture was stirred for 2 h at 65 °C. After removal of the solvent under reduced pressure, the residue was purified by preparative HPLC, using a C-18 reversed-phase column (CH_3_CN/H_2_O/TFA 25:75:0.1). HCl solution (1 M) was added and the solvent was removed by freeze drying yielding a pink solid (60.0 mg, 44%). Mp 272–276 °C; ^1^H NMR (CD_3_OD, 400 MHz) 8.39 (s, 1H), 8.21 (s, 1H), 4.18 (m, 4H), 3.94 (t, ^3^*J* = 6.2 Hz, 1H), 3.61 (t, ^3^*J* = 6.9 Hz, 2H), 3.46 (m, 4H), 3.06 (s, 18H), 2.17 (m, 4H), 2.04-1.53 (m, 6H); ^13^C NMR (CD_3_OD, 101 MHz) 171.9, 167.1, 163.6, 163.5, 162.7, 153.1, 135.2, 133.5, 129.5, 128.8, 124.8, 123.2, 122.6, 121.8, 100.8, 65.7, 54.0, 53.9, 53.8, 53.8, 43.9, 39.2, 38.6, 31.4, 30.2, 23.8, 23.4, 23.3; HRMS-ESI^+^ (methanol, *m*/*z*): [M]^2+^ calcd for C_32_H_45_ClN_6_O_6_, 322.1545; found, 322.1551; UV–vis (cacodylate buffer, pH 5.0) λ [nm] (ε [M^−1^ cm^−1^]) 540 (9200), 370 (8800), 352 (7700), 335 (5600); fluorescence (cacodylate buffer, pH 5.0, λ_ex_ = 470 nm): λ_max_ [nm] = 602; Φ_fl_ = 0.10.

**Synthesis of *N,N'*-bis((3-(dimethylamino)propyl)amino)-2-(2-(dimethylamino)ethyl)amino-6-chloro-1,4,5,8-naphthalenetetracarboxylic acid diimide (4):** 2-(Dimethylamino)ethylamine (52.0 mg, 0.59 mmol, 64.5 μL) was added to a solution of diimide (**1**, 100 mg, 198 μmol) in CH_3_CN (16 mL) and the reaction mixture was stirred for 4 h at 70 °C under nitrogen. After removal of the solvent under reduced pressure, the residue was purified by column chromatography (CH_2_Cl_2_/MeOH/NEt_3_ 93:7:0.1 → 90:10:0.1). Solvent evaporation and drying yielded a red solid (66.0 mg, 60%). Mp: 164–168 °C; ^1^H NMR (CDCl_3_, 400 MHz) 10.13 (t, *^3^J* = 5 Hz, 1H), 8.51 (s, 1H), 8.19 (s, 1H), 4.19 (m, 4H), 3.62 (m, 2H), 2.72 (m, 2H), 2.43 (m, 4H), 2.37 (s, 6H), 2.25 (s, 12H), 1.89 (m, 4H); ^13^C NMR (CDCl_3_, 101 MHz) 165.6, 162.1, 162.0, 161.3, 151.5, 134.8, 132.9, 128.1, 127.2, 123.7, 121.8, 121.1, 120.9, 100.1, 58.0, 57.3, 57.3, 45.6, 45.5, 41.3, 39.7, 38.8, 26.0, 25.9.; HRMS-ESI^+^ (methanol, *m*/*z*): [M + H]^+^ calcd for C_28_H_38_ClN_6_O_4_, 557.26431; found, 557.26419; UV–vis (MeOH) λ [nm] (ε [M^−1^ cm^−1^]) 524 (10800), 364 (9500), 347 (8200), 329 (6400).

**Synthesis of *N,N*'-di((3-(trimethylammonium)propyl)amino)-2-(2-(trimethylammonium)ethyl)amino-6-chloro-1,4,5,8-naphthalenetetracarboxylic acid diimide trichloride (5):** To a solution of diimide (**4**, 90.0 mg, 161 μmol) in acetonitrile (16 mL) was added iodomethane (77.8 mg, 34.0 μL, 548 μmol). The reaction mixture was stirred for 3 d at room temperature. After removal of the solvent under reduced pressure, the residue was purified by preparative HPLC, using a C-18 reversed-phase column (CH_3_CN/H_2_O/TFA 20:80:0.1). HCl solution (1 M) was added and the solvent was removed by freeze drying yielding a red solid (50.0 mg, 44%). Mp 263 °C; ^1^H NMR (CD_3_OD, 400 MHz) 8.61 (s, 1H), 8.40 (s, 1H), 4.28 (m, 6H), 3.84 (t, ^3^*J* = 6.8 Hz, 2H), 3.54 (m, 4H), 3.34 (s, 9H), 3.14 (s, 18H), 2.27 (m, 4H); ^13^C NMR (D_2_O, 101 MHz) 165.3, 162.46, 162.42, 161.5, 150.9, 134.0, 132.9, 127.2, 126.6, 122.9, 120.9, 120.6, 120.2, 100.6, 63.9, 53.6, 53.0, 53.0, 52.9, 38.0, 37.4, 36.8, 21.4, 21.3; HRMS-ESI^+^ (methanol, *m*/*z*): [M]^3+^ calcd for C_31_H_46_ClN_6_O_4_, 200.4423; found, 200.4423; UV–vis (cacodylate buffer, pH 5.0) λ [nm] (ε [M^−1^ cm^−1^]) 518 (9400), 368 (9800), 350 (7900), 332 (5100); fluorescence (cacodylate buffer, pH 5.0, λ_ex_ = 470 nm): λ_max_ [nm] = 573; Φ_fl_ = 0.32.

### Spectrophotometric studies

The UV–vis spectra were recorded on a Varian Cary 100 Bio spectrophotometer or on a Jasco V670/770 spectrometer, steady state fluorescence spectra were measured on a PTI QM4/2003 or Varian Eclipse spectrofluorimeter and CD spectra on JASCO J815 spectrophotometer at 25 °C using appropriate 1 cm path quartz cuvettes. For study of interactions with DNA and RNA, aqueous solutions of compounds buffered to pH 5.0 (buffer sodium cacodylate, I = 0.05 mol dm^−3^) were used. The fluorescence quantum yields were determined by the optically dilute method (A < 0.05) by using *N*,*N*′-di(*n*-octyl)-2-chloro-6-*n*-octylamino-1,4,5,8-naphthalenetetracarboxylic acid diimide (Φ_fl_ = 0.58 in CH_2_Cl_2_) as standard [25,59]. The reported quantum yields are averaged values obtained at four different excitation wavelengths for each NDI.

Polynucleotides were purchased as noted: poly(dAdT)– poly(dAdT), poly(dGdC)-poly(dGdC), poly(A)–poly(U) (Sigma-Aldrich. St. Louis. USA), calf thymus (ct)-DNA (Aldrich). Polynucleotides were dissolved at pH 7 according to manufacturer instructions and diluted 1000–10000 times in pH 5 buffer of same type (Na-cacodylate buffer, I = 0.05 M). The calf thymus ct-DNA was additionally sonicated and filtered through a 0.45 mm filter [60]. The polynucleotide concentration was determined spectroscopically as the concentration of nucleo bases (equimolar to phosphates).

Fluorimetric titrations were performed at pH 5.0 (I = 0.05 mol dm^−3^, buffer sodium cacodylate) by adding portions of polynucleotide solution into the solution of the studied compound and CD experiments were done by adding portions of compound stock solution into the solution of polynucleotide. Titration data were processed by the Scatchard equation [40–42]. Values for *K*_s_ and *n* given in Table 3 all have satisfactory correlation coefficients (>0.999). Thermal melting curves for DNA, RNA, and their complexes with the studied compounds (Table 2) were determined as previously described [39,61] by following the absorption change at 260 nm as a function of temperature. Absorbance of the ligands was subtracted from every curve and the absorbance scale was normalized. The *T*_m_ values are the midpoints of the transition curves determined from the maximum of the first derivative and checked graphically by the tangent method [59]. The Δ*T*_m_ values were calculated by subtracting the *T*_m_ value of the free nucleic acid from the *T*_m_ value of the complex. Every Δ*T*_m_ value here reported was the average of at least two measurements. The error in Δ*T*_m_ is ±0.5 °C.

In a similar manner as described in [62] ITC were carried out at 293 K on a MicroCal VP-iTC instrument. In the ITC titration experiments aliquots of the compounds (28 × 10 µL, *c* = 0.10–0.15 mM) were injected from a 280 µL rotating syringe (307 rpm) into the calorimeter reaction cell containing 1.4406 mL of the corresponding polynucleotides (*c* = 0.05–0.8 mM). Blank experiments were carried out to determine the heats of dilution of the compounds and the polynucleotides. All solutions used in the ITC experiments were degassed under vacuum prior to use to eliminate air bubbles.

## Supporting Information

File 1 Copies of ^1^H, ^13^C NMR spectra, HRMS spectra, and titration data on the binding of water-soluble NDI compounds **3a**,**b** and **5** with DNA/RNA.
