# Supplementary material for: Naphthalene diimide–amino acid conjugates as novel fluorimetric and CD probes for differentiation between ds-DNA and ds-RNA
Source: Beilstein J Org Chem. 2020 Aug 19;16:2032–45. doi: 10.3762/bjoc.16.170 (PMC7445415; doi:10.3762/bjoc.16.170)
Supplement: File 1 — Copies of 1H, 13C NMR spectra, HRMS spectra, and titration data on the binding of water-soluble NDI compounds 3a,b and 5 with DNA/RNA. [file Beilstein_J_Org_Chem-16-2032-s001.pdf]

BEILSTEIN JOURNAL OF ORGANIC CHEMISTRY

Supporting Information

for

Naphthalene diimide–amino acid conjugates as novel fluorimetric and CD probes for differentiation between ds-DNA and ds-RNA

Annike Weißenstein, Myroslav O. Vysotsky, Ivo Piantanida and Frank Würthner

Beilstein J. Org. Chem. **2020,** *16,* 2032–2045. doi:10.3762/bjoc.16.170

Copies of ^{1}H, ^{13}C NMR spectra, HRMS spectra, and titration data on the binding of water-soluble NDI compounds 3a,b and 5 with DNA/RNA

License and Terms: This is a supporting information file under the terms of the Creative Commons Attribution License (https://creativecommons.org/licenses/by/4.0). Please note that the reuse, redistribution and reproduction in particular requires that the authors and source are credited.
The license is subject to the *Beilstein Journal of Organic Chemistry* terms and conditions: (https://www.beilstein-journals.org/bjoc)

Table of contents

1.	^{1}H, ^{13}C NMR spectra for the new dye compounds	S3
2.	HRMS spectra for the new dye compounds	S8
3.	Melting studies of polyA-polyU with NDIs **3a**,**b** and **5**	S10
4.	Fluorescence titrations of NDIs **3a**,**b** and **5** with different DNA/RNA	S11
5.	CD titrations of NDIs **3a**,**b** and **5** with different DNA/RNA	S15
6.	Calorimetric titrations (ITC) of NDIs **3a**,**b** and **5** with different DNA/RNA	S18

1. ^{1}H, ^{13}C NMR spectra for the new dye compounds

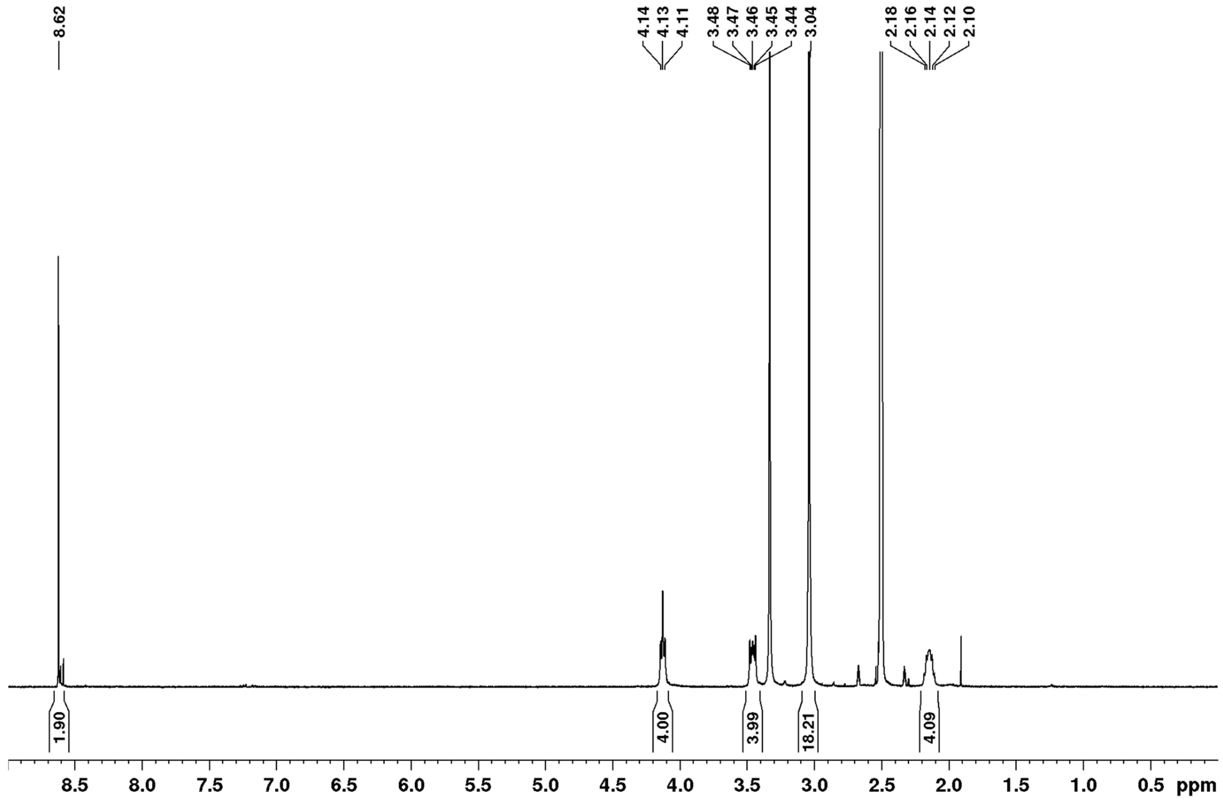

Figure S1. ^{1}H NMR spectrum (400 MHz, DMSO-d_6, 298 K) of NDI **2**.

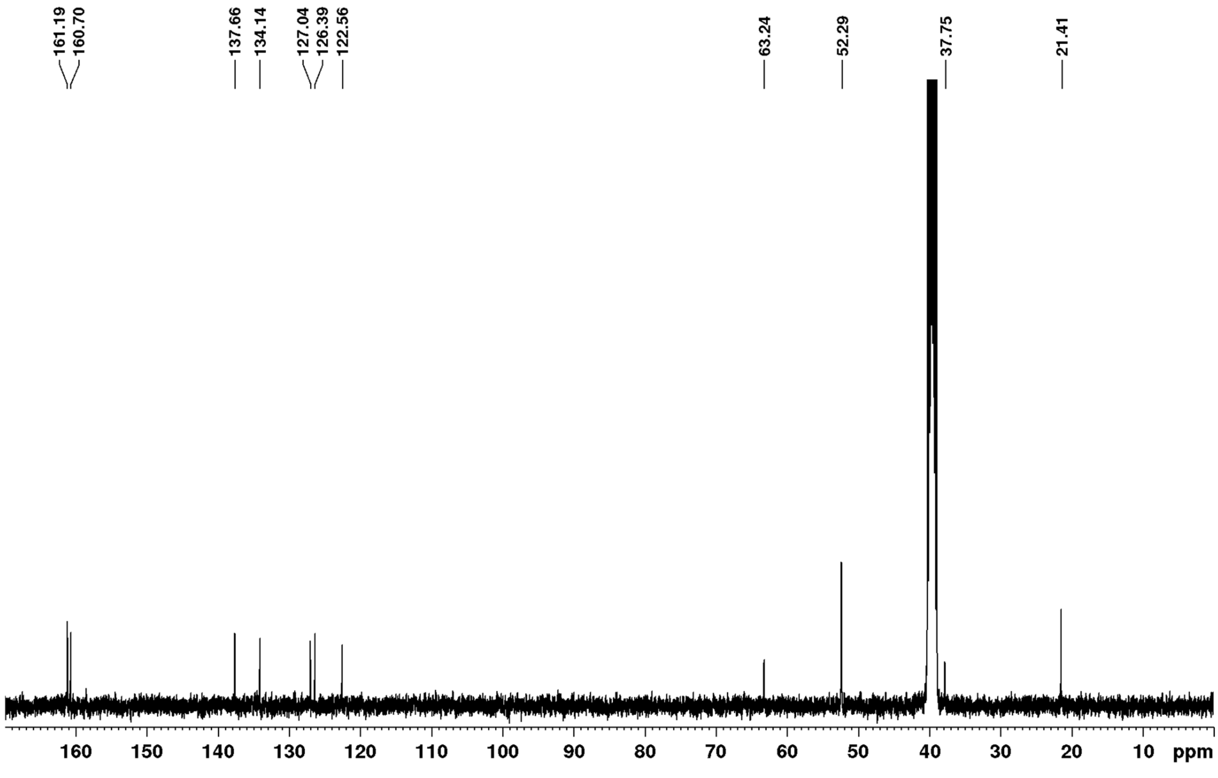

Figure S2. ^{13}C NMR spectrum (101 MHz, DMSO-d_6, 298 K) of NDI **2**.

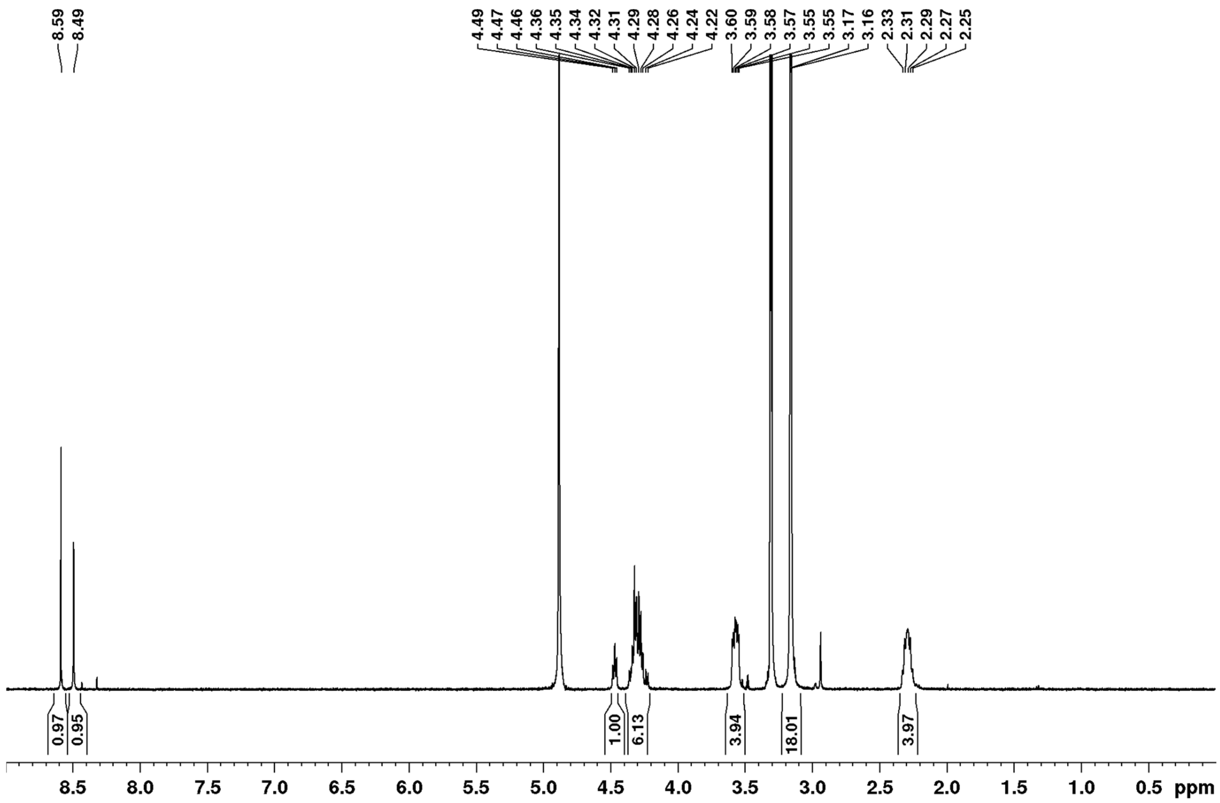

Figure S3. ^{1}H NMR spectrum (400 MHz, CD_3OD, 298 K) of NDI **3a**.

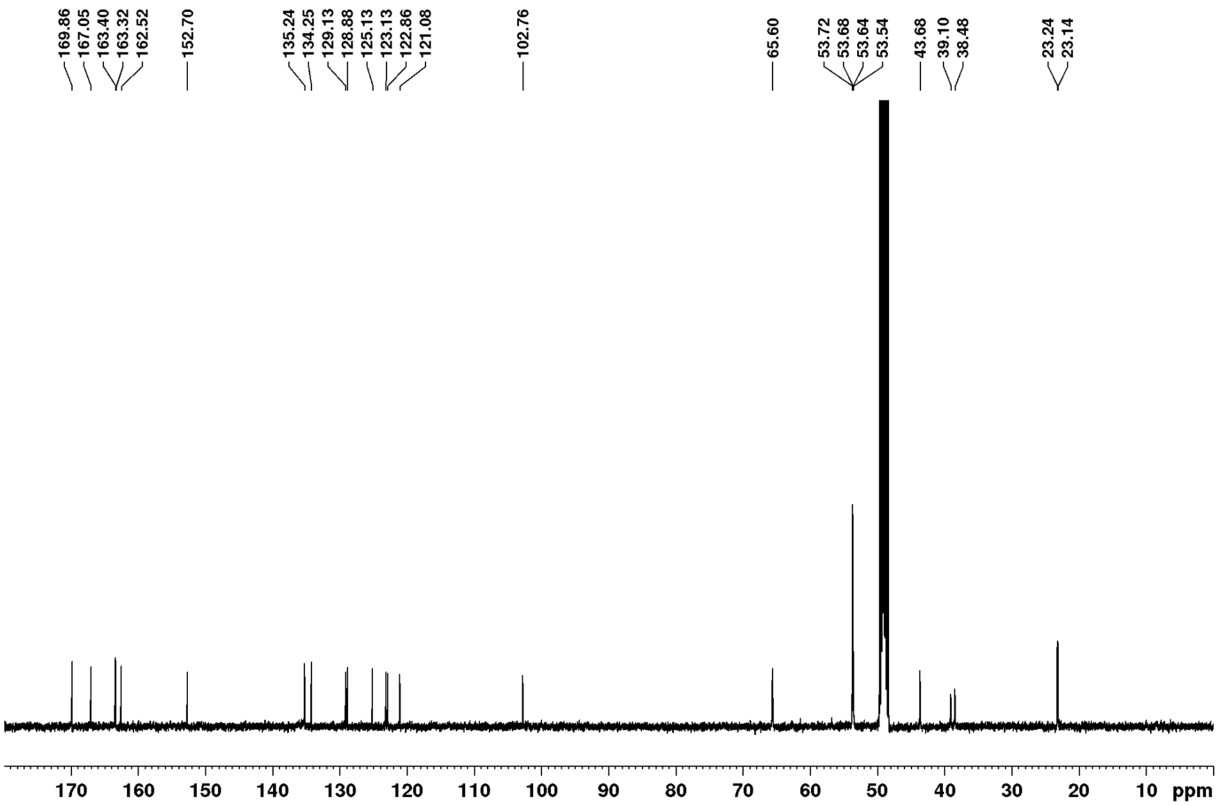

Figure S4. ^{13}C NMR spectrum (101 MHz, CD_3OD, 298 K) of NDI **3a**.

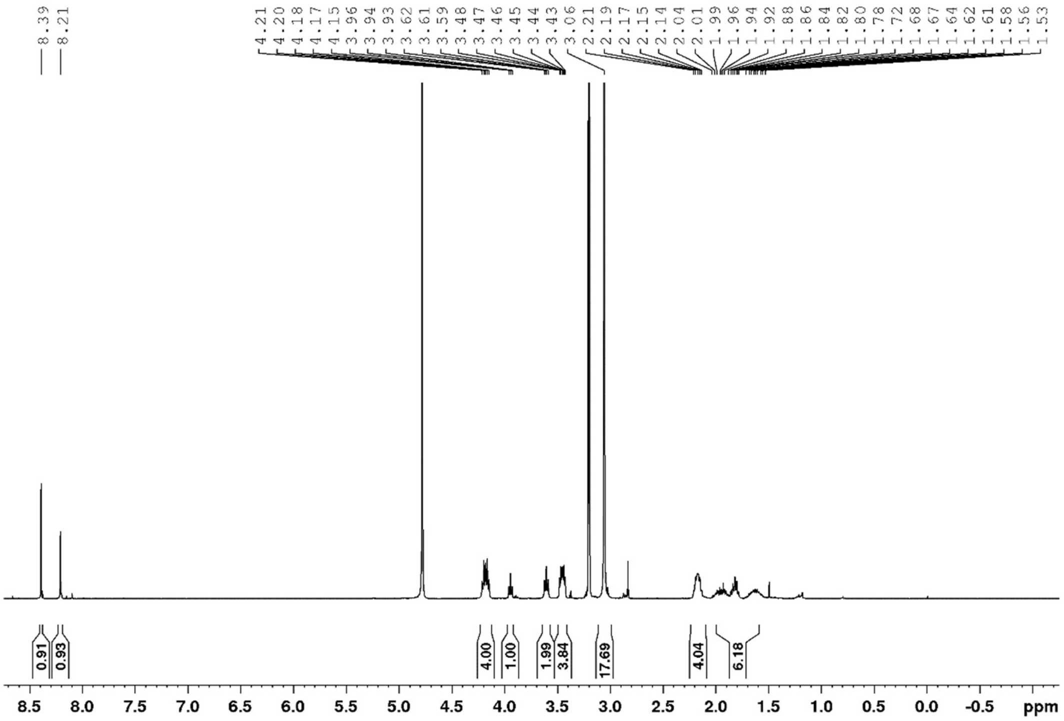

Figure S5. ^{1}H NMR spectrum (400 MHz, CD_3OD, 298 K) of NDI **3b**.

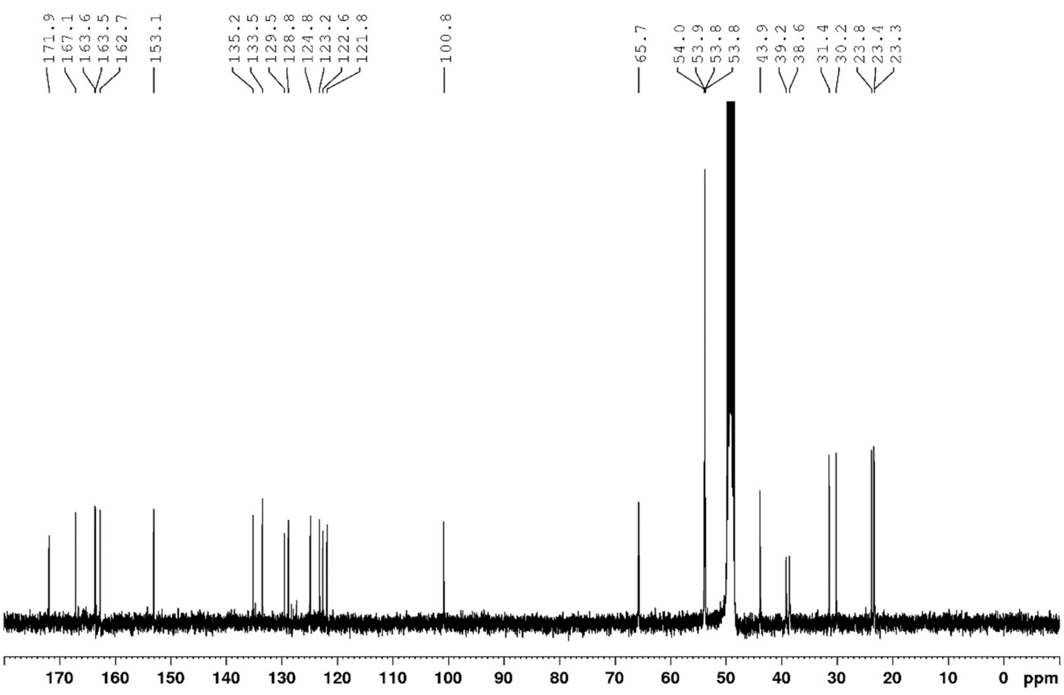

Figure S6. ^{13}C NMR spectrum (101 MHz, CD_3OD, 298 K) of NDI **3b**.

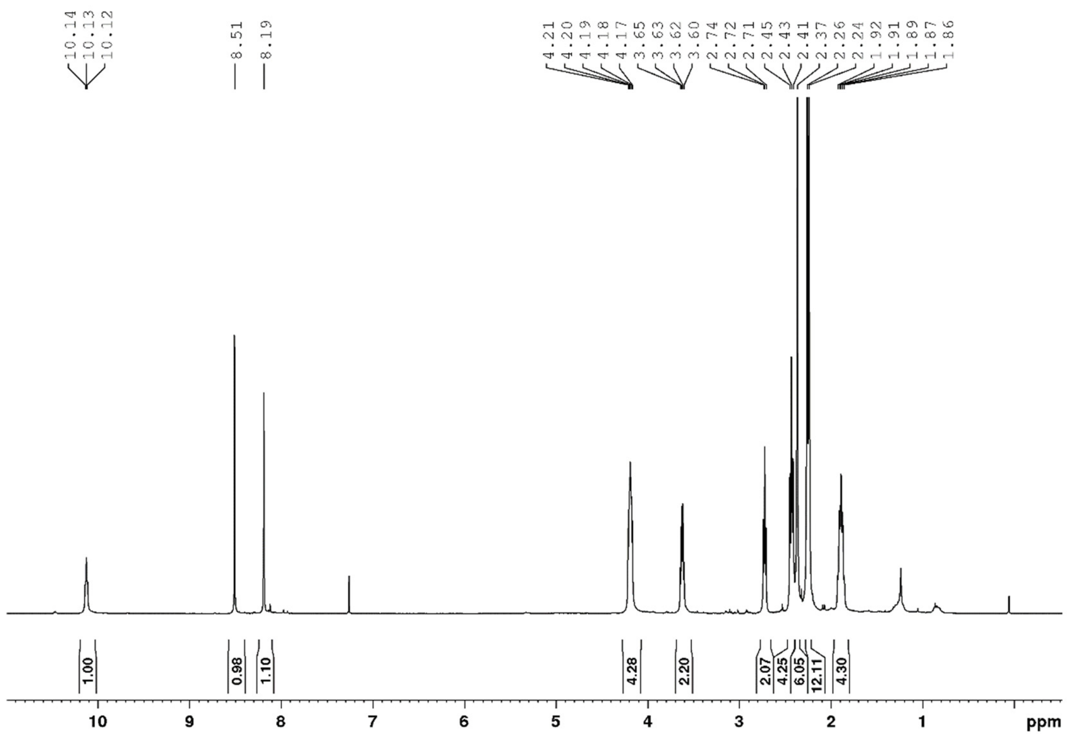

Figure S7. ^{1}H NMR spectrum (400 MHz, $CDCl_3$, 298 K) of NDI **4**.

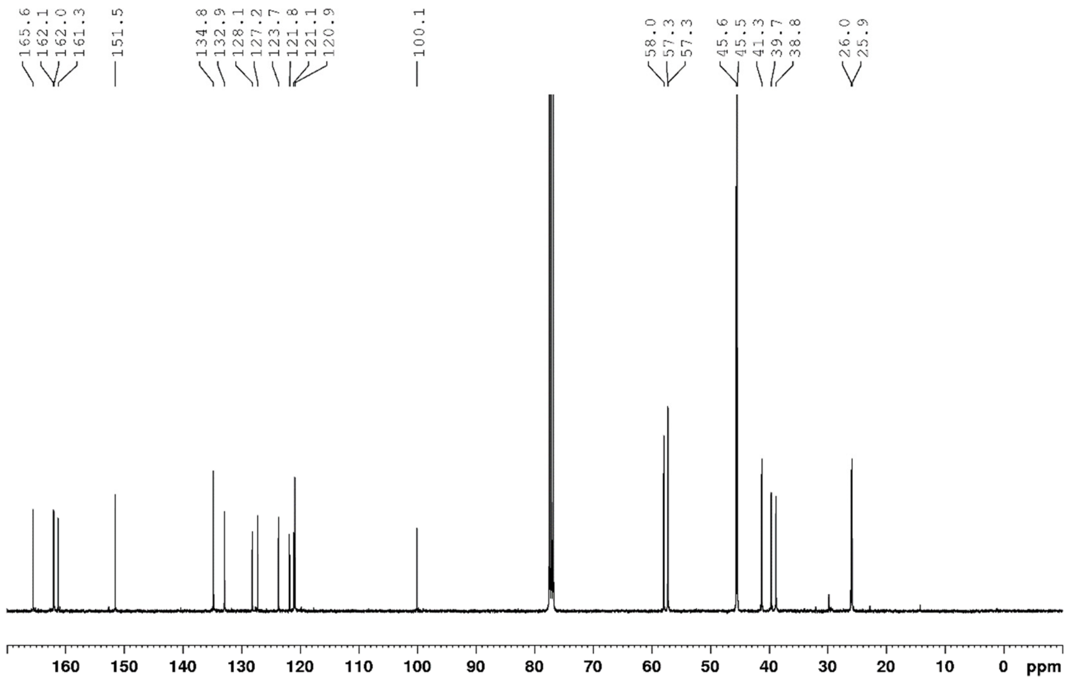

Figure S8. ^{13}C NMR spectrum (101 MHz, $CDCl_3$, 298 K) of NDI **4**.

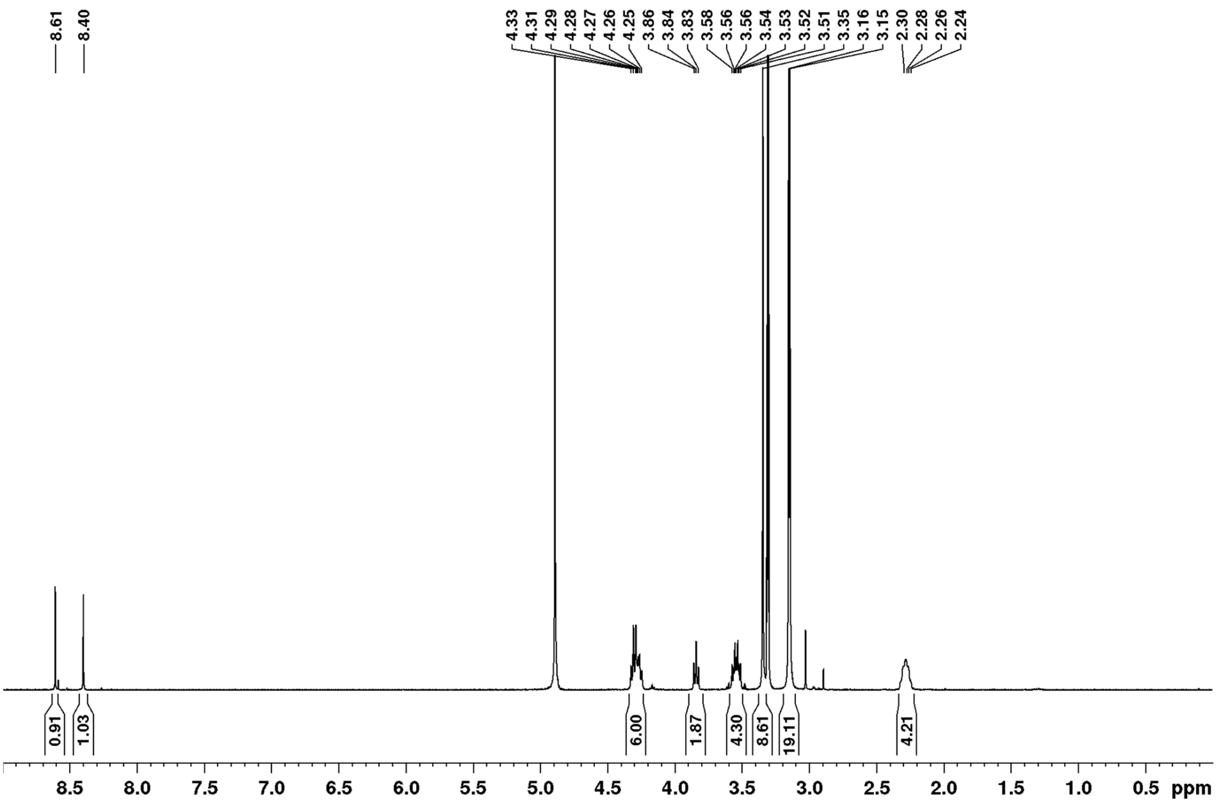

Figure S9. ^{1}H NMR spectrum (400 MHz, CD_3OD, 298 K) of NDI **5**.

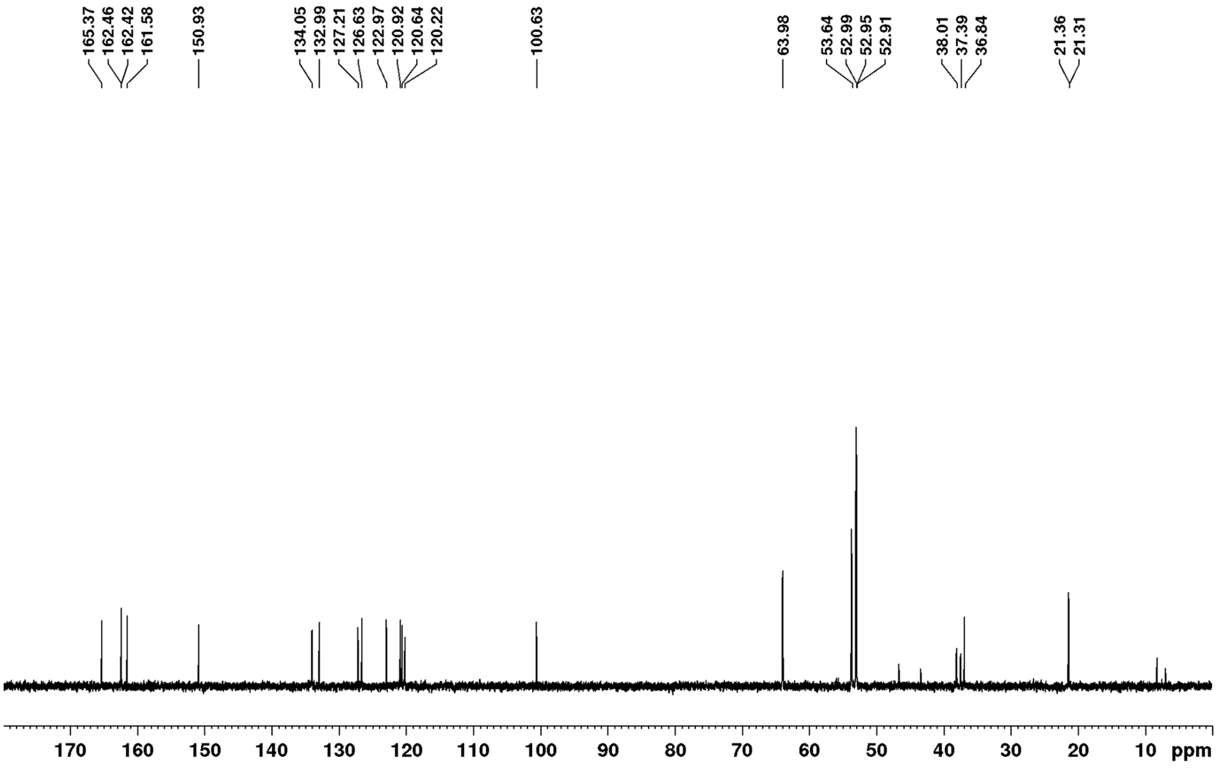

Figure S10. ^{13}C NMR spectrum (101 MHz, D_2O, 298 K) of NDI **5**.

2. HRMS spectra for the new dye compounds

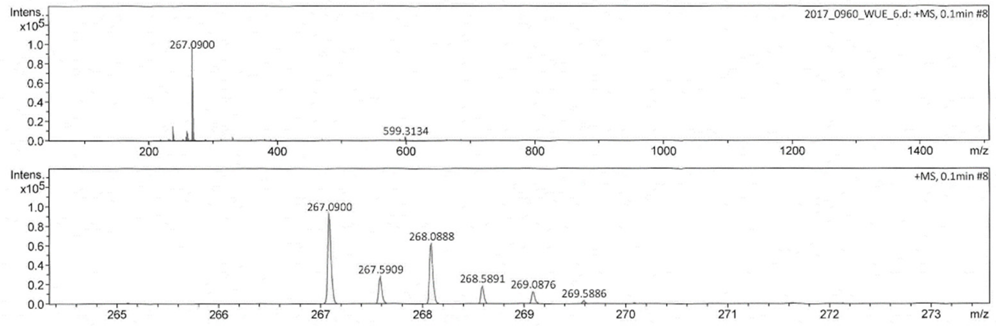

Meas. m/z	#	Ion Formula	m/z	err [ppm]	mSigma	# mSigma	Score	rdb	e⁻ Conf	N-Rule
267.0900	1	C26H32Cl2N4O4	267.0895	-1.9	9.9	1	100.00	12.0	even	ok

Figure S11. High-resolution mass spectrum (ESI) of NDI **2**.

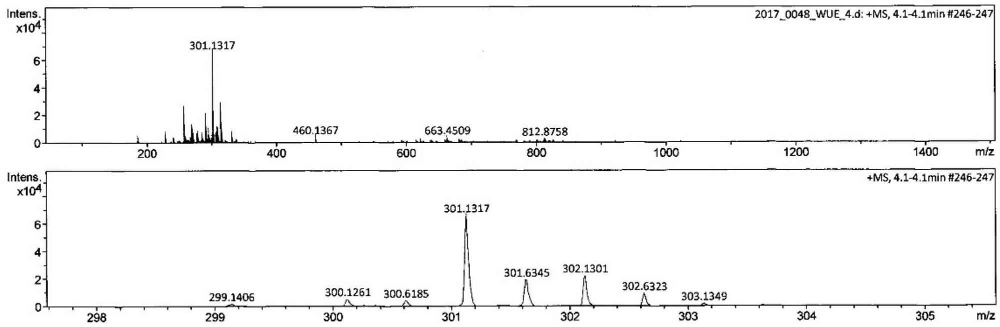

Meas. m/z	#	Ion Formula	m/z	err [ppm]	mSigma	# mSigma	Score	rdb	e⁻ Conf	N-Rule
301.1317	1	C29H39ClN6O6	301.1304	-4.1	36.9	1	100.00	13.0	even	ok

Figure S12. High-resolution mass spectrum (ESI) of NDI **3a**.

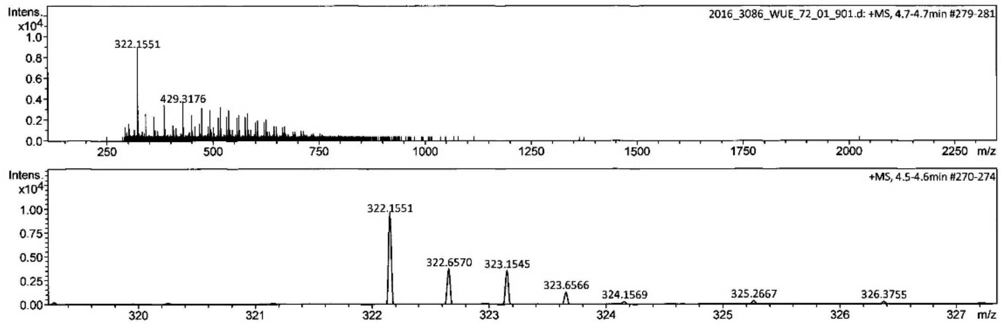

Meas. m/z	#	Ion Formula	m/z	err [ppm]	mSigma	# mSigma	Score	rdb	e⁻ Conf	N-Rule
322.1551	1	C32H45ClN6O6	322.1539	-3.6	14.2	1	100.00	13.0	even	ok

Figure S13. High-resolution mass spectrum (ESI) of NDI **3b**.

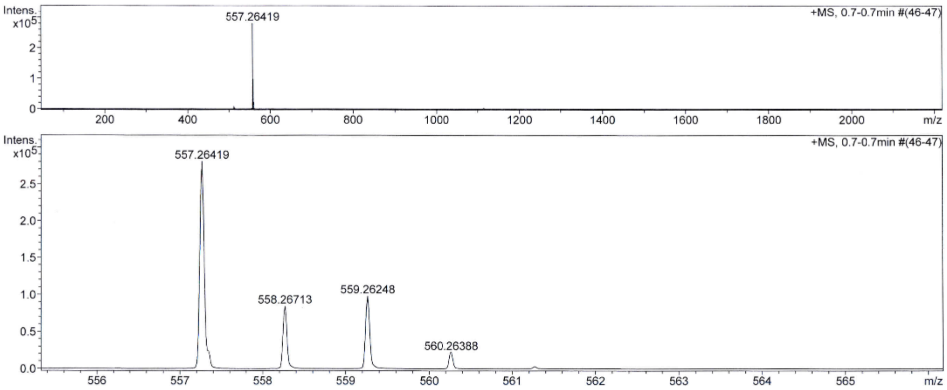

Figure S14. High-resolution mass spectrum (ESI) of NDI **4**.

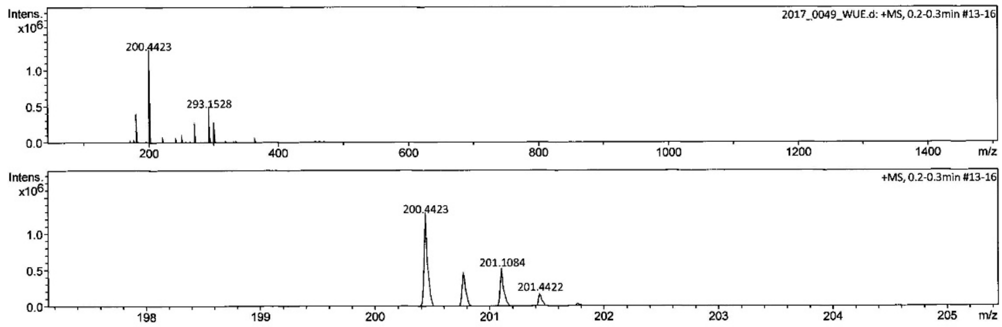

Meas. m/z	#	Ion Formula	m/z	err [ppm]	mSigma	# mSigma	Score	rdb	e⁻ Conf	N-Rule
200.4423	1	C31H46ClN6O4	200.4418	-2.8	4.1	1	100.00	11.5	even	ok

Figure S15. High-resolution mass spectrum (ESI) of NDI **5**.

3. Melting studies of polyA-polyU with NDIs 3a,b and 5

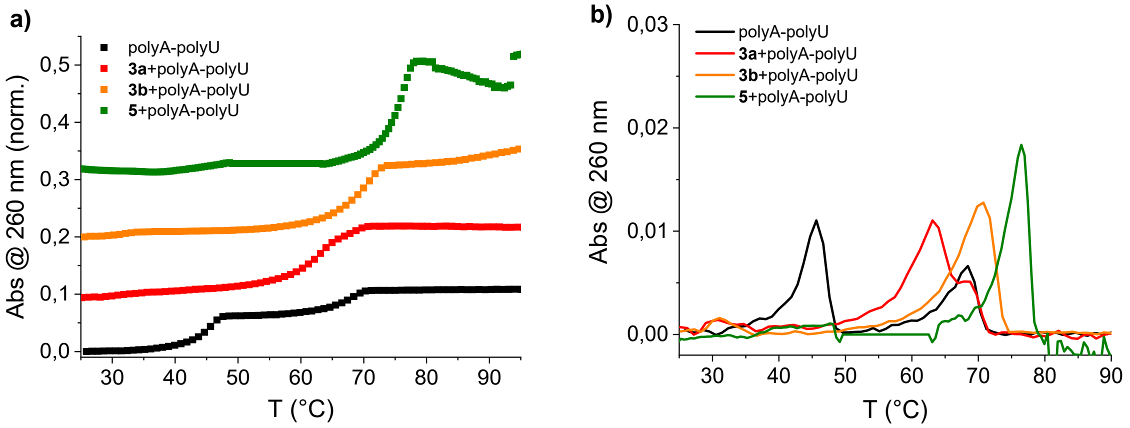

Figure S16. (a) Thermal denaturation profiles of polyA-polyU with the addition of **3a**,**b** and **5** (*r* = 0.3 ([NDI] / [polynucleotide])) (sodium cacodylate buffer, pH 5.0, I = 0.05 M); (b) First derivative function of absorption from temperature. Note biphasic transition of free poly A-poly U: the first transition at T_m = 47.3 °C is attributed to denaturation of poly A-poly U and the second transition at T_m = 71.1 °C is attributed to denaturation of poly AH^+-poly AH^+ since poly A at pH 5.0 is mostly protonated and forms ds-polynucleotide.[1]

4. Fluorescence titrations of NDIs 3a,b and 5 with different DNA/RNA

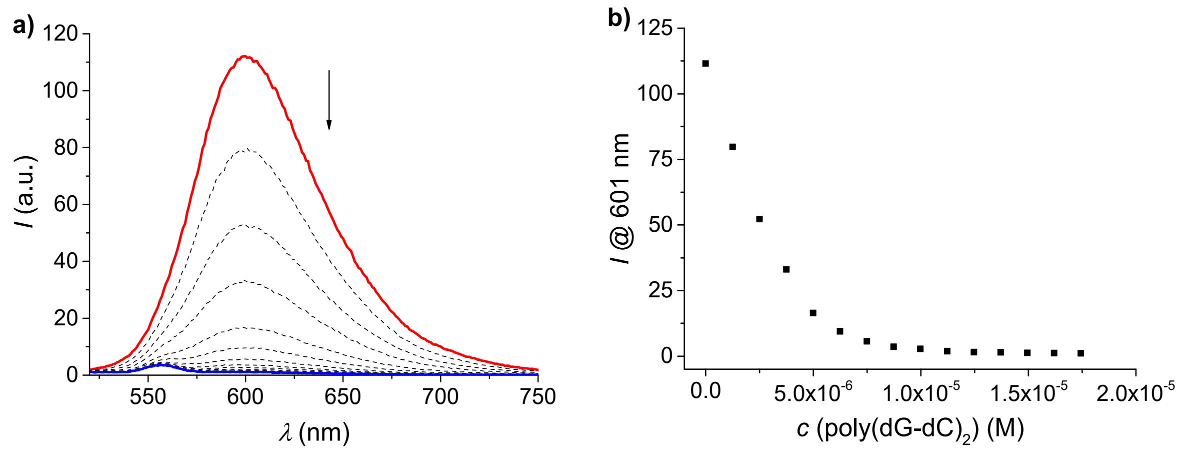

Figure S17. (a) Changes in the fluorescence spectra of **3b** ($c = 1.00 \times 10^{-6}$ M, λ_{ex} = 470 nm) with the addition of poly(dG-dC)$_2$ ($c = 1.25 \times 10^{-6}$ - 1.74×10^{-5} M) in cacodylate buffer, pH 5.0 at 25 ° C. (b) Dependence of the fluorescence intensity of **3b** at λ_{max} = 601 nm on c (poly(dG-dC)$_2$) (cacodylate buffer, pH 5.0, I = 0.05 M).

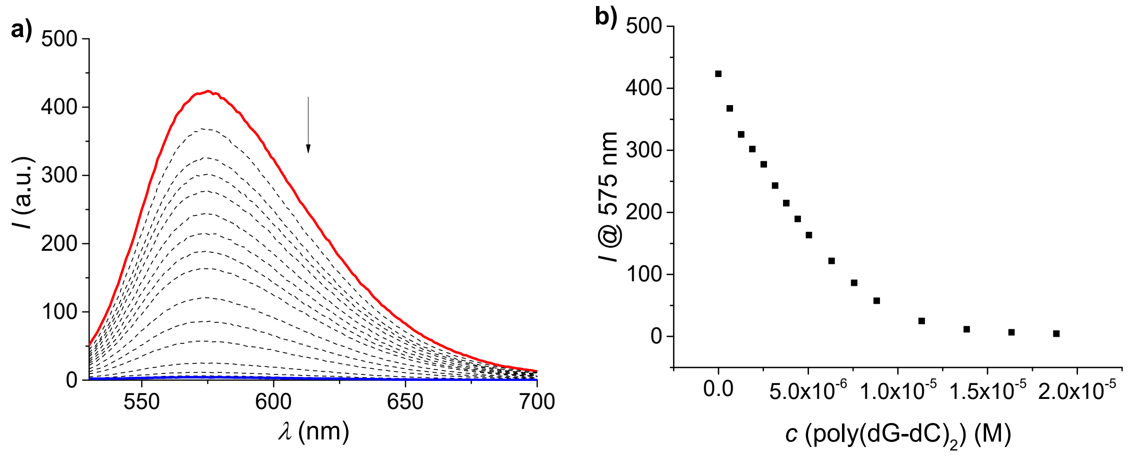

Figure S18. (a) Changes in the fluorescence spectra of **5** ($c = 1.00 \times 10^{-6}$ M, λ_{ex} = 470 nm) with the addition of poly(dG-dC)$_2$ ($c = 6.32 \times 10^{-7}$ – 1.88×10^{-5} M) in cacodylate buffer pH 5.0 at 25 ° C. (b) Dependence of the fluorescence intensity of **5** at λ_{max} = 575 nm on c (poly(dG-dC)$_2$) (cacodylate buffer pH 5.0, I = 0.05 M).

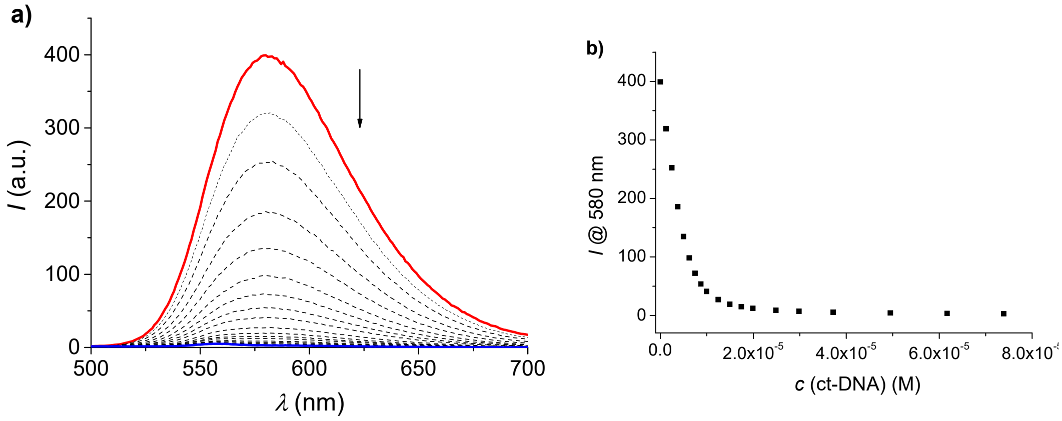

Figure S19. (a) Changes in the fluorescence spectra of **3a** ($c = 1.00 \times 10^{-6}$ M, λ_{ex} = 470 nm) with the addition of ct-DNA ($c = 1.24 \times 10^{-6} - 7.38 \times 10^{-5}$ M) in cacodylate buffer pH 5.0 at 25 °C. (b) Dependence of the fluorescence intensity of **3a** at λ_{max} = 580 nm on c (ct-DNA) (cacodylate buffer pH 5.0, I = 0.05 M).

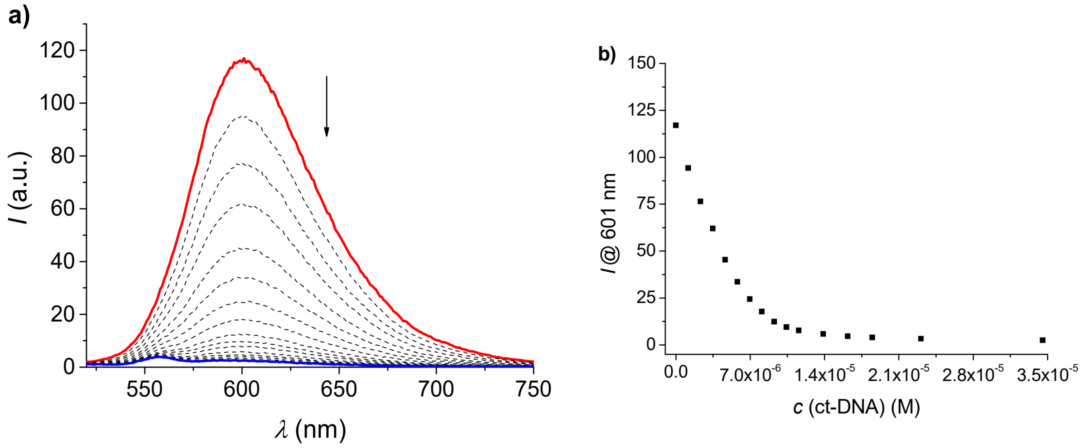

Figure S20. (a) Changes in the fluorescence spectra of **3b** ($c = 1.00 \times 10^{-6}$ M, λ_{ex} = 470 nm) with the addition of ct-DNA ($c = 1.15 \times 10^{-6} - 3.45 \times 10^{-5}$ M) in cacodylate buffer pH 5.0 at 25 °C. (b) Dependence of the fluorescence intensity of **3b** at λ_{max} = 601 nm on c (ct-DNA) (cacodylate buffer pH 5.0, I = 0.05 M).

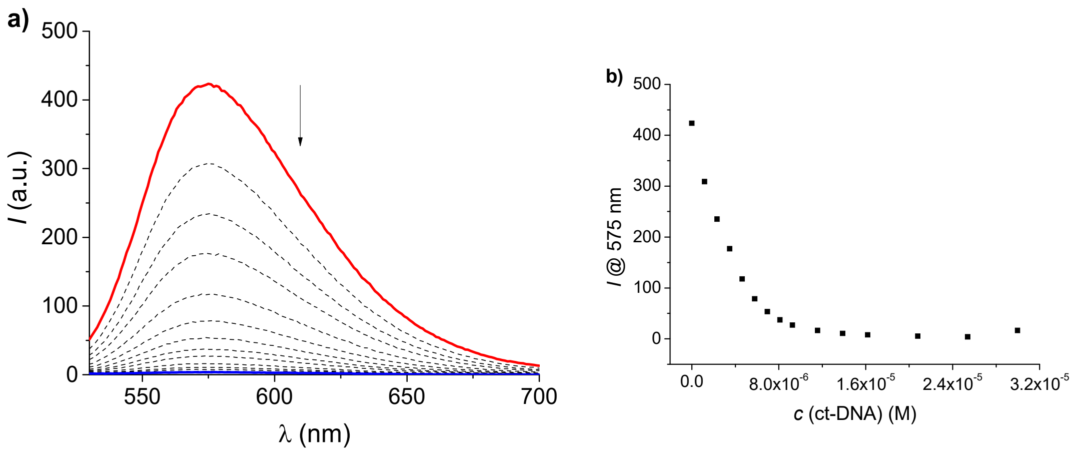

Figure S21. (a) Changes in the fluorescence spectra of **5** (c = 1.00 x 10^{-6} M, λ_{ex} = 470 nm) with the addition of ct-DNA (c = 1.15 x 10^{-6} – 2.99 x 10^{-5} M) in cacodylate buffer pH 5.0 at 25 °C. (b) Dependence of the fluorescence intensity of **5** at λ_{max} = 575 nm on c(ct-DNA) (cacodylate buffer pH 5.0, I = 0.05 M).

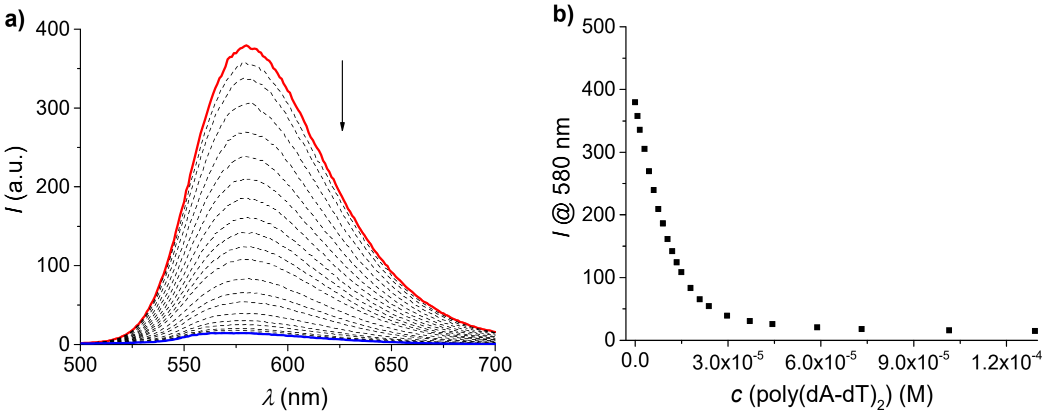

Figure S22. (a) Changes in the fluorescence spectra of **3a** (c = 1.00 x 10^{-6} M, λ_{ex} = 470 nm) with the addition of poly(dA-dT)$_2$ (c = 7.50 x 10^{-7} – 1.29 x 10^{-4} M) in cacodylate buffer pH 5.0 at 25 °C. (b) Dependence of the fluorescence intensity of **3a** at λ_{max} = 580 nm on c(poly (dA-dT)$_2$) (cacodylate buffer pH 5.0, I = 0.05 M).

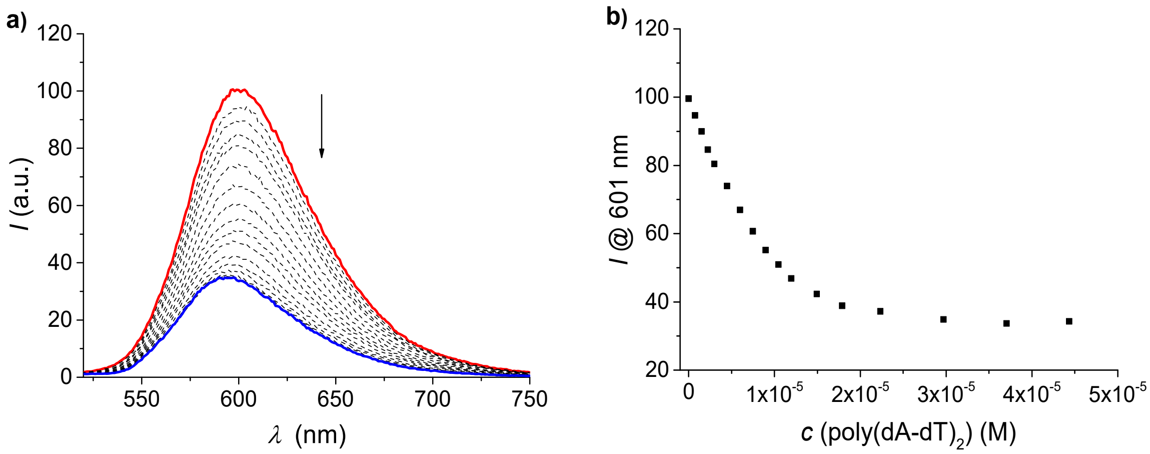

Figure S23. (a) Changes in the fluorescence spectra of **3b** ($c = 1.00 \times 10^{-6}$ M, λ_{ex} = 470 nm) with the addition of poly(dA-dT)$_2$ ($c = 7.50 \times 10^{-7} – 4.43 \times 10^{-5}$ M) in cacodylate buffer pH 5.0 at 25 °C. (b) Dependence of the fluorescence intensity of **3b** at λ_{max} = 601 nm on c(poly (dA-dT)$_2$) (cacodylate buffer pH 5.0, I = 0.05 M).

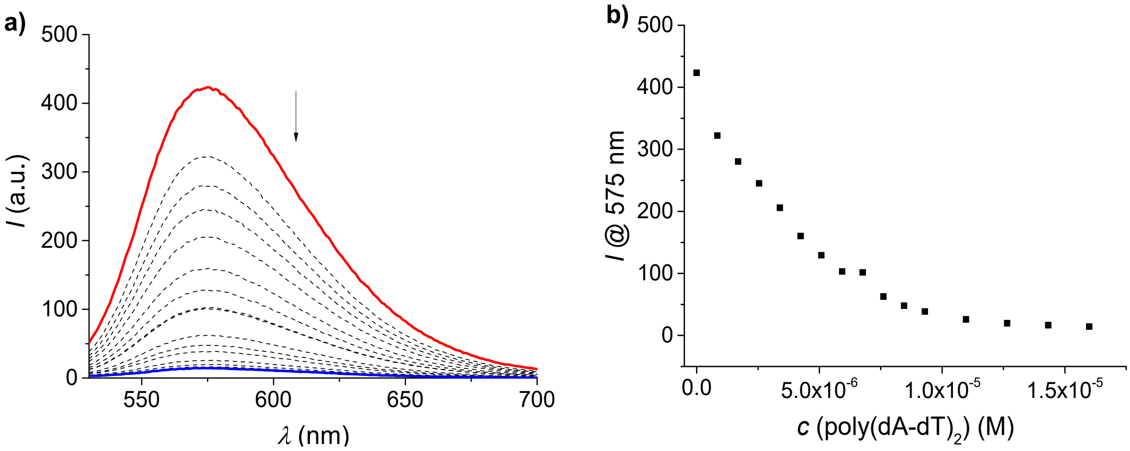

Figure S24. (a) Changes in the fluorescence spectra of **5** ($c = 1.00 \times 10^{-6}$ M, λ_{ex} = 470 nm) with the addition of poly(dA-dT)$_2$ ($c = 8.49 \times 10^{-7} – 1.59 \times 10^{-5}$ M) in cacodylate buffer pH 5.0 at 25 °C. (b) Dependence of the fluorescence intensity of **5** at λ_{max} = 575 nm on c(poly(dA-dT)$_2$) (cacodylate buffer, pH 5.0, I = 0.05 M).

5. CD titrations of NDIs 3a,b and 5 with different DNA/RNA

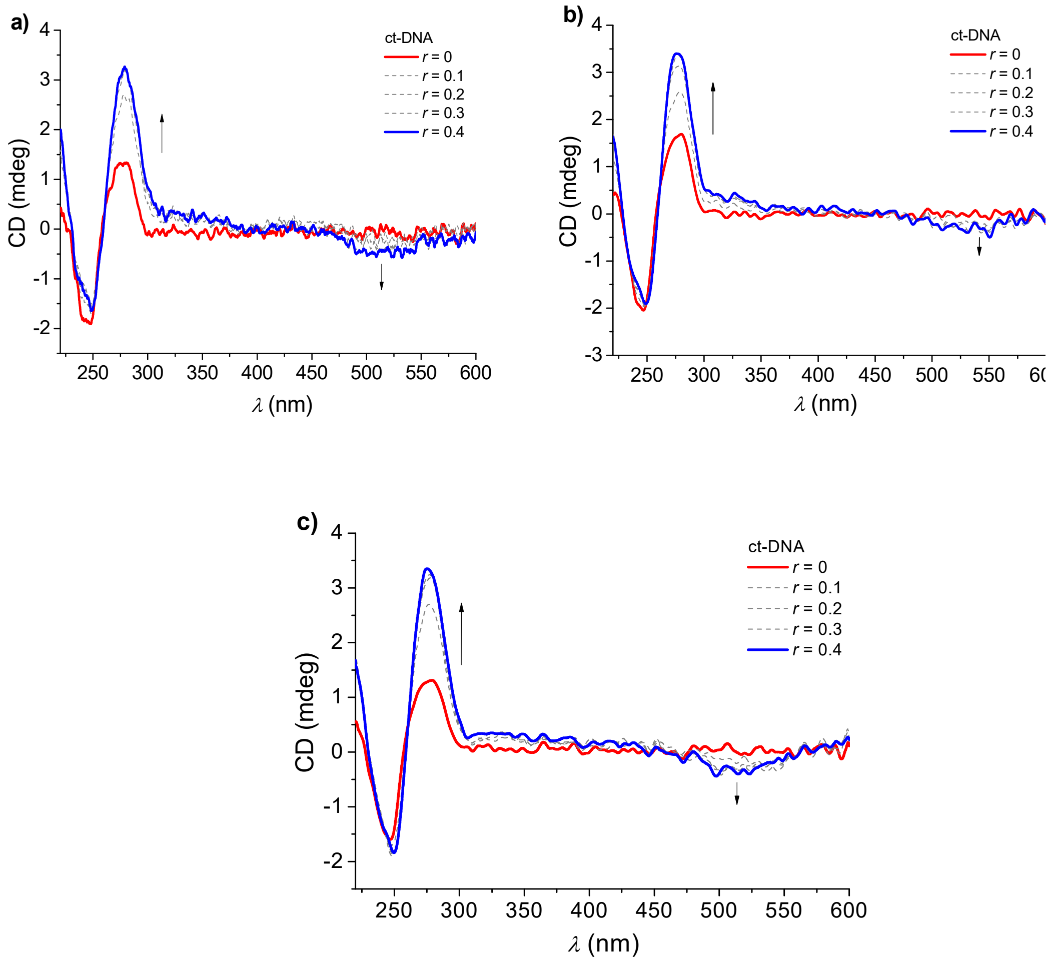

Figure S25. CD titration of NDI (a) **3a**, (b) **3b** and (c) **5** with ct-DNA (c = 2.00 x 10^{-5} M) at molar ratios of r = [NDI]/[polynucleotide] at 25 ° C (cacodylate buffer, pH 5.0, I = 0.05 M).

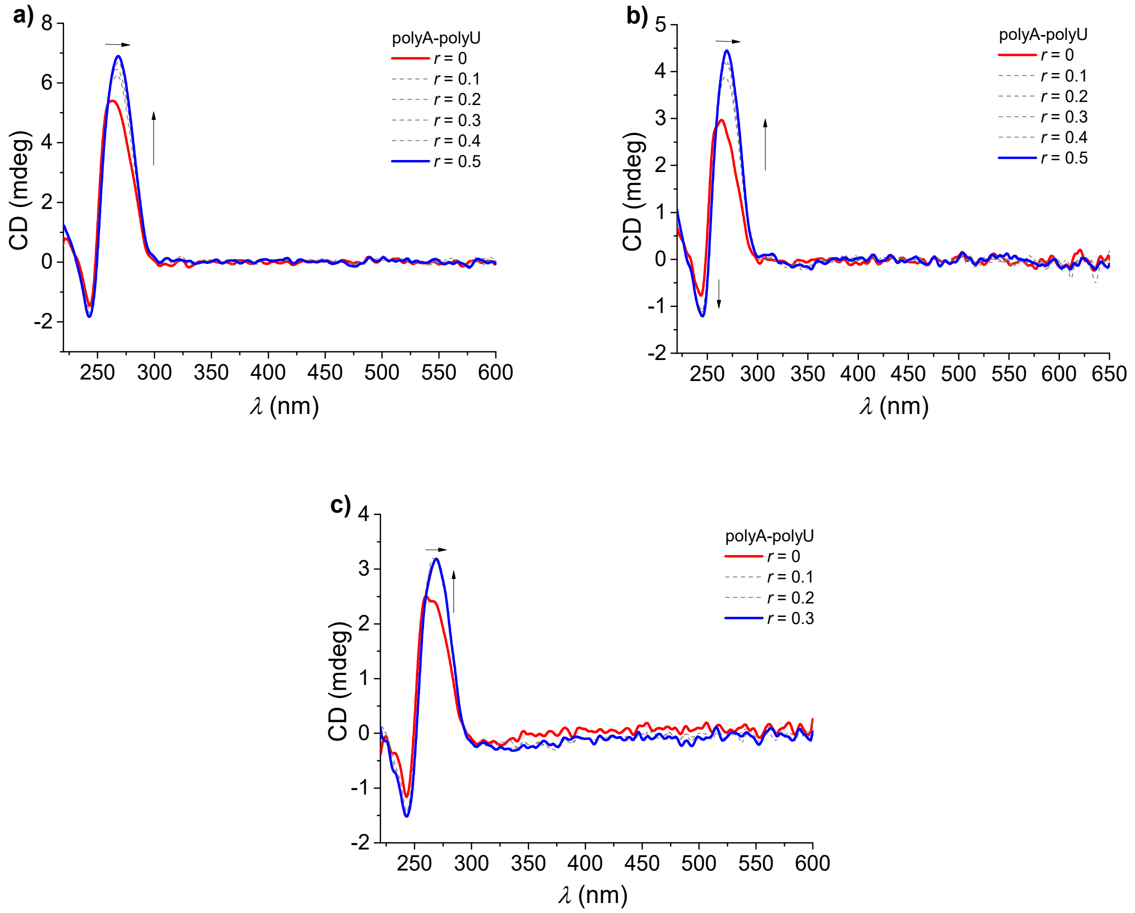

Figure S26. CD titration of NDI (a) **3a**, (b) **3b** and (c) **5** with polyA-polyU (c = 2.00 x 10^{-5} M) at molar ratios of *r* = [NBI]/[polynucleotide] at 25 °C (cacodylate buffer, pH 5.0, I = 0.05 M).

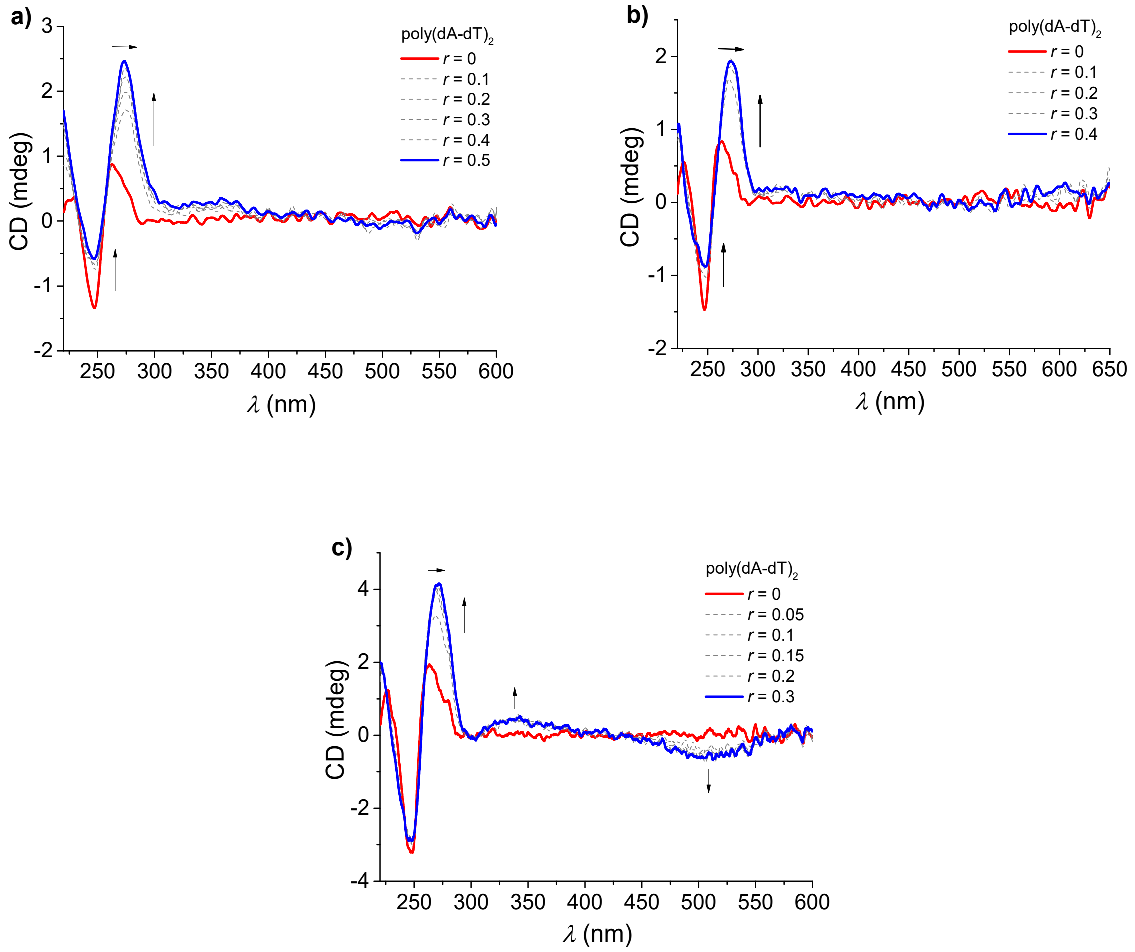

Figure S27. CD titration of NDI (a) **3a**, (b) **3b** and (c) **5** with poly(dA-dT)2 (c = 2.00 x 10^{-5} M) at molar ratios of *r* = [NBI]/[polynucleotide] at 25 °C (cacodylate buffer, pH 5.0, I = 0.05 M).

6. Calorimetric titrations (ITC) of NDIs 3a,b and 5 with different DNA/RNA

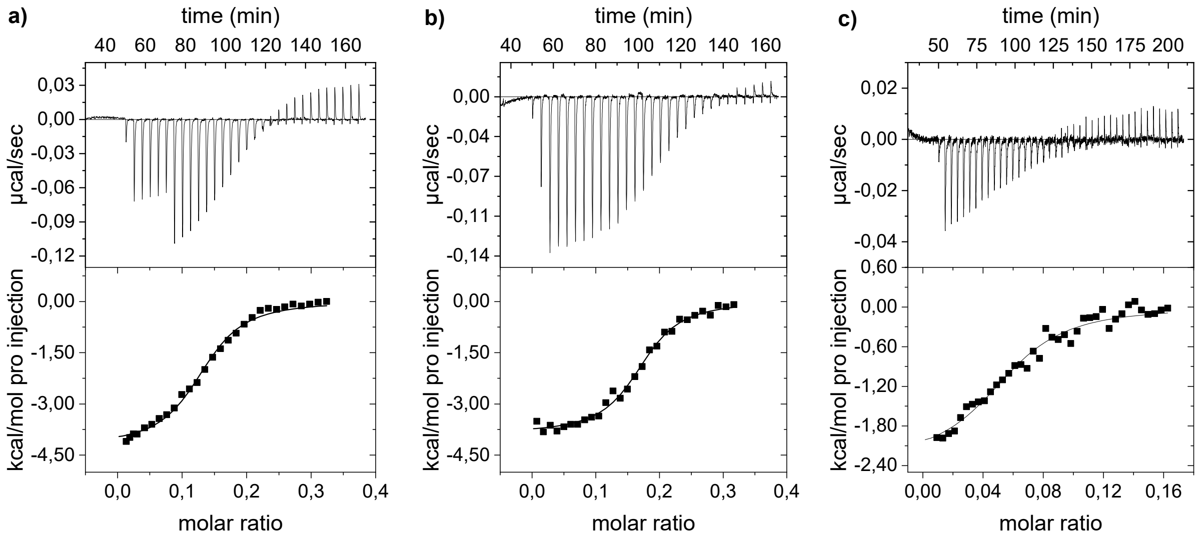

Figure S28. Calorimetric titrations of (a) ct-DNA, (b) poly(dA-dT)$_2$ and (c) polyA-polyU solution in cacodylate buffer at 298 K with NDI **3a**. Top: data obtained after periodic injection of an NDI **3a** solution; bottom: graph of heat/mol released per injection versus the molar ratio of NDI **3a** to polynucleotide.

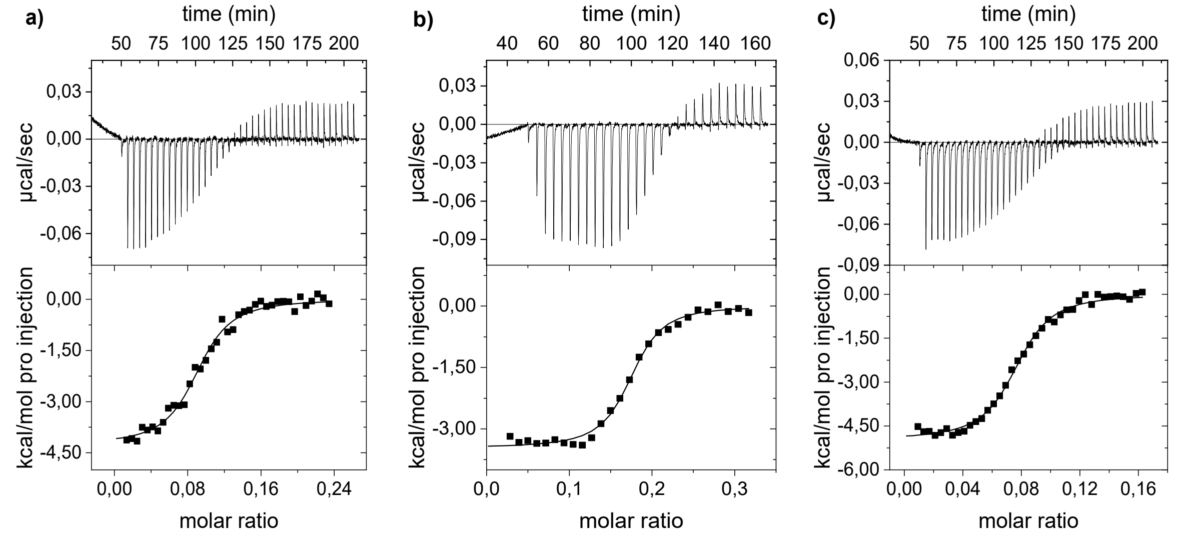

Figure S29. Calorimetric titrations of (a) ct-DNA, (b) poly(dA-dT)$_2$ and (c) polyA-polyU solution in cacodylate buffer at 298 K with NDI **3b**. Top: data obtained after periodic injection of an NDI **3b** solution; bottom: graph of heat/mol released per injection versus molar ratio of NDI **3b** to polynucleotide.

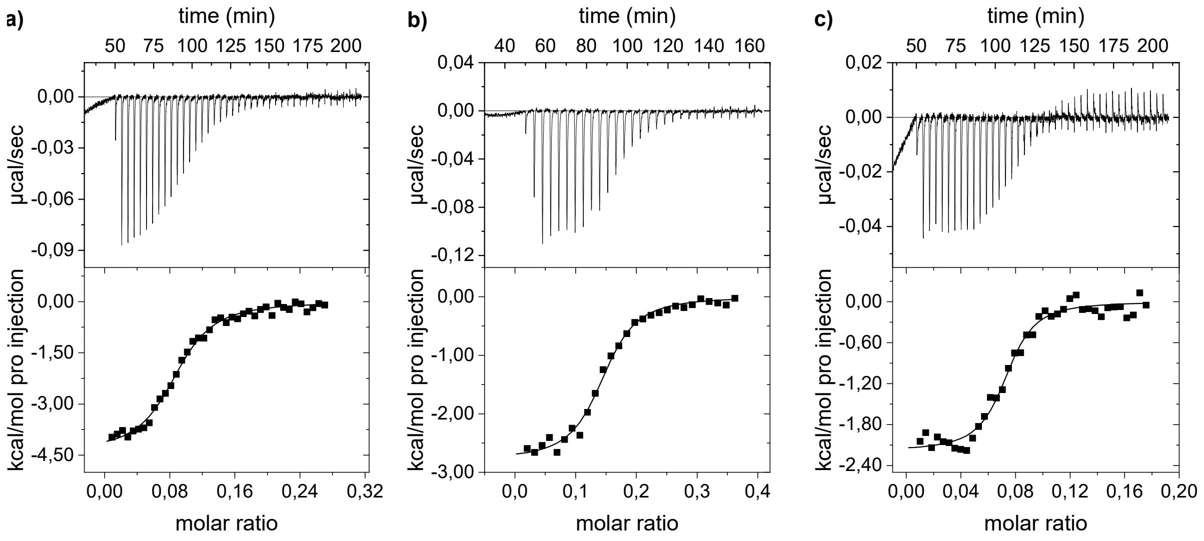

Figure S30. Calorimetric titrations of (a) ct-DNA, (b) poly(dA-dT)$_2$ and (c) polyA-polyU solution in cacodylate buffer at 298 K with NDI **5**. Top: data obtained after periodic injection of an NDI **5** solution; bottom: graph of heat/mol released per injection versus molar ratio of NDI **5** to polynucleotide.

---

[1] Cantor, C. R.; Schimmel, P. R. *Biophysical Chemistry*; WH Freeman and Co., San Francisco: 1980; Vol. 3,
